# A High-Resolution Rotational Analysis of 2‑Chloroethanol (HOCH_2_CH_2_Cl) and Its First Vibrationally Excited State

**DOI:** 10.1021/acsearthspacechem.6c00221

**Published:** 2026-09-21

**Authors:** Hayley A. Bunn, Holdson H. Liang, Brian J. Esselman, Paul L. Raston, Andi Pracko, Steven Shipman, Brant Billinghurst, Jianbao Zhao, Jes K. Jørgensen, Susanna L. Widicus Weaver

**Affiliations:** † Department of Chemistry, 5228 University of Wisconsin−madison, Madison, Wisconsin 53706, United States; ‡ Department of Chemistry, University of Hawai'i at Ma̅noa, Honolulu, Hawaii 96822, United States; § Department of Chemistry, 10648 New College of Florida, Sarasota, Florida 34243, United States; ∥ BrightSpec, Inc., 770 Harris St., Suite 104b, Charlottesville, Virginia 22903-4583, United States; ⊥ Canadian Light Source. Inc., University of Saskatchewan institution, Saskatoon, Saskatchewan S7N 2 V3, Canada; # Niels Bohr Institute, University of Copenhagen, Jagtvej 155A, Copenhagen N., Copenhagen DK-2200, Denmark; ∇ Department of Astronomy, University of Wisconsin−Madison, Madison, Wisconsin 53706, United States

**Keywords:** Rotational Spectroscopy, High-Resolution Infrared Spectroscopy, Nuclear Quadrupole Coupling, Astronomical Search

## Abstract

An extensive analysis of the high-resolution rotational spectrum (8–26 GHz, 140–700 GHz, 100–600 cm^–1^) of the simplest stable chlorohydrin, 2-chloroethanol, is provided by combining high-resolution infrared, microwave, and millimeter-wave spectra. The ground state and first vibrationally excited state of both chlorine isotopologues (HOCH_2_CH_2_^35^Cl and HOCH_2_CH_2_^37^Cl) are well treated using a single-state distorted rotor Hamiltonian in the A reduction and I^*r*^ representation, reporting up to a full set of octic distortion constants. The extended microwave spectrum collected and the well-resolved quadrupole splitting observed therein allowed for the further refinement of quadrupole coupling parameters. Inclusion of rotationally resolved infrared transitions enabled the accurate and precise determination of the vibrational band origin of ν_21_ for HOCH_2_CH_2_^35^Cl (150.908 599 5 (43) cm^–1^). The high spectral density made measurement and least-squares fitting of transitions in both rotational and high-resolution infrared spectra challenging and is the likely source of the larger-than-expected σ_fit_ value of up to 0.060 MHz for each fit. Nevertheless, the extended millimeter-wave spectrum reported here provides spectral frequencies that directly overlap with astronomical data, allowing for a more thorough search for this species in the interstellar medium. Despite its expected formation from HCl with either oxirane or ethylene glycol in the interstellar medium, 2-chloroethanol continues to evade detection. We report upper limits on the column density toward 30 astrophysically diverse regions. The reported upper limits do not provide significant new constraints on the chemistry of this species in the interstellar medium and highlight the limited knowledge of interstellar chlorine chemistry.

## Graphical abstract

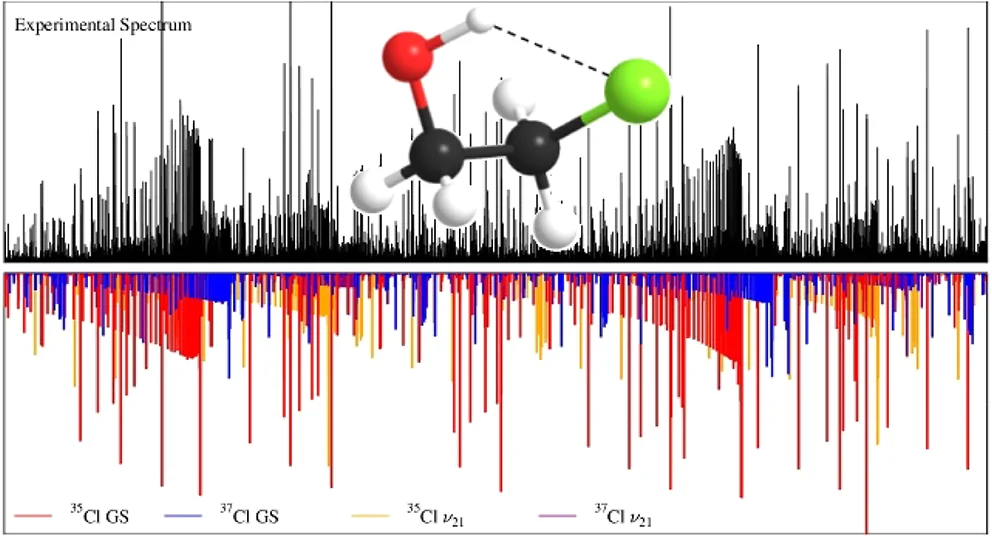

## Introduction

Halogenated hydrocarbons are emitted by industrial, biological, and geological processes. They are particularly well known for their problematic effect on atmospheric ozone. Due to their potential industrial and biological origins, these species have been proposed as possible biomarkers in the search for life on other planets.^1–3^ Chlorine species have been observed in carbonaceous chondrites^4^ and were detected on Mars by the Curiosity rover.^5,6^ According to the Cologne Database of Molecular Spectroscopy,^7^ five neutral molecules containing chlorine have been confirmed in the interstellar medium including four diatomic species, with HCl first observed in 1985 toward OMC-1,^8^ AlCl,^9^ NaCl, and KCl first observed toward IRC+10216 in 1987,^9^ and one organohalogen (CH_3_Cl) observed toward IRAS16293-2422 B in 2017.^4^ Our understanding of halogen chemistry in the interstellar medium is, therefore, rather limited. Chemical models predict that hydrogen chloride accounts for at least 90% of the chlorine in the interstellar medium^10^ and its reaction with other known interstellar constituents, *i.e*., oxirane, *c*-(CH_2_)_2_O, or ethylene glycol, (CH_2_OH)_2_, is predicted to form 2-chloroethanol (HOCH_2_CH_2_Cl) by reaction in cosmic-ray processed interstellar ices.^11^ 2-Chloroethanol is known to pyrolytically decompose into HCl and vinyl alcohol (CH_2_CHOH), which subsequently tautomerizes to acetaldehyde (CH_3_CHO).^12^ All of these species are well known in the interstellar medium. The recent search for 2-chloroethanol toward Sgr B2­(N) using the previously reported molecular constants resulting from a least-squares fit of microwave and rotationally resolved infrared transitions was unsuccessful.^11^ The large partition function of 2-chloroethanol results in a weak spectrum, making its detection more difficult than that of smaller molecules. The limited available laboratory rotational spectra for 2-chloroethanol, however, further hampers the chances of detecting this species. The lack of a detection in the previous search and the ambiguity in the transition frequencies highlights the need for measurement of the 2-chloroethanol spectrum across a broader portion of the frequency range of modern telescopes.

2-Chloroethanol exists as five distinct conformers, several of which form enantiomeric pairs.^13,14^ The *Gg*′ conformer, designating the *gauche* internal rotation around both the C–C bond and the C–O bond, respectively, is the lowest in energy by 1.50 kcal/mol (CCSD/cc-pVDZ).^13,14^ Unlike the other conformers, *Gg*′ is likely stabilized by a hydrogen-bond-like interaction between the hydroxyl hydrogen and the chlorine (Figure 1). The next lowest-energy conformer, *Ag,* has an enantiomer that favors its spectral intensity via statistical doubling like *Gg*′, however, only the *Gg*′ conformer has been reported in gas-phase high-resolution spectra (microwave and infrared).^11,15,16^ A very thorough structural and spectral analysis is reported in the microwave region (<40 GHz) for nine isotopologues of the lowest-energy conformer.^15,16^ Those works report accurate ground-state rotational and distortion constants along with quadrupole coupling parameters. Spectroscopic constants for the first three excited fundamental states (ν_21_, ν_20_, ν_19_) and the first overtone (2ν_21_) are also reported.

**Figure 1:**
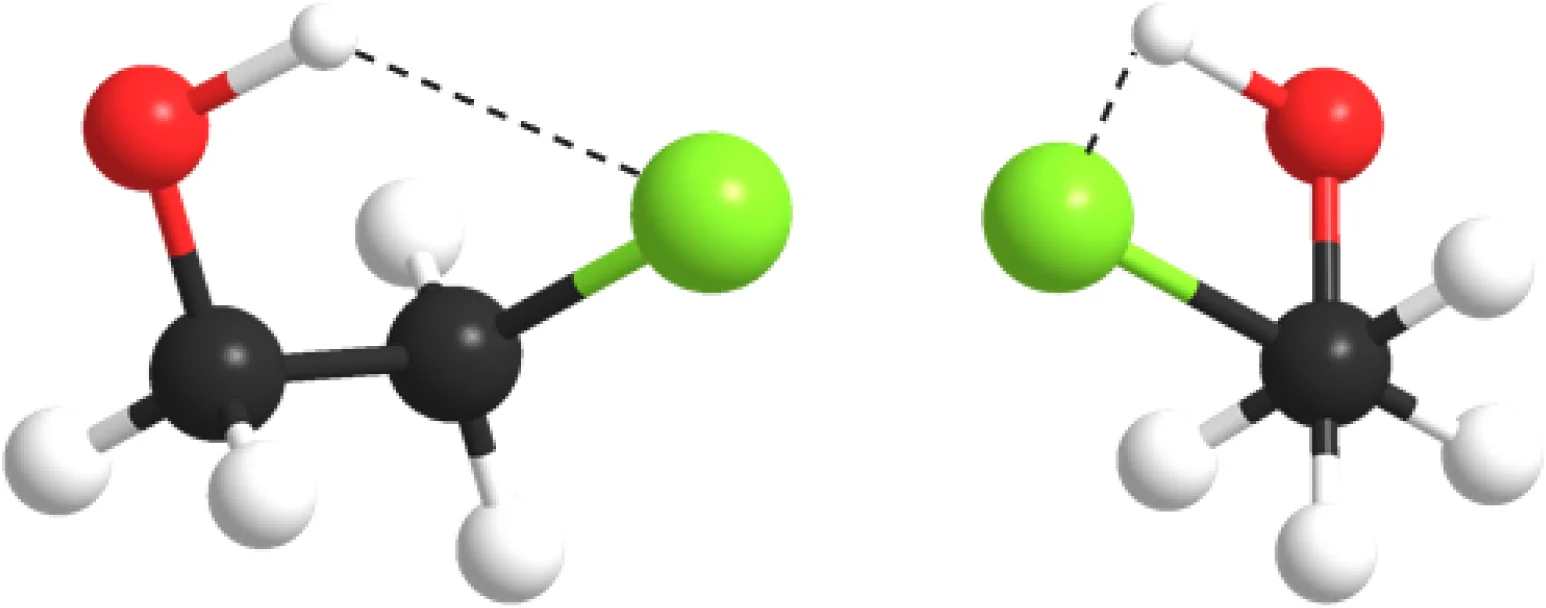
*Gg′* conformer of 2-chloroethanol from two perspectives. *G*/*g* refers to a *gauche* conformation, with the upper-case symbol for the C–C rotation and the lower-case symbol for the C–O bond rotation. The *Gg′* conformer is stabilized by an intramolecular hydrogen bond interaction.

More recently, synchrotron-based high-resolution far-infrared spectroscopy has been increasingly applied to the study of low frequency vibrations in astrochemically relevant molecules, including hydrogen-bonded alcohols and haloalcohols.^17^ In one of those works the measurement, analysis, and least squares fitting of the high resolution far infrared spectrum of 2-chloroethanol was reported, including several vibrational modes below 350 cm^–1^.^11,17,18^ A fit to the rotational structure of the ν_19_ vibrational mode combined with the previous microwave work resulted in improved ground-state spectroscopic constants for both chlorine isotopologues and well-refined ν_19_ excited-state spectroscopic constants. A fit of the ν_19+21_ ← ν_21_ hot band resulted in excited-state constants for both vibrational states, whereas all other observed bands have lower intensity or are highly congested due to overlap of multiple vibrational modes.

Here, we provide an extension to the high-resolution rotational work, reporting the spectrum of the most stable *Gg′* conformer of 2-chloroethanol for both chlorine isotopologues in the microwave (MW; 8–26 GHz), millimeter (mm; 140–700 GHz) and infrared (IR; 100–600 cm^–1^) frequencies. A thorough analysis of the ground state and first vibrationally excited state for each isotopologue of chlorine is provided, along with precise rotational constants and centrifugal distortion constants up to the octic level. Analysis of the newly acquired microwave spectra also allows for a further refinement of the quadrupole coupling constants in these isotopologues. In the millimeter spectrum, we observe rotational transitions arising from more than seven additional vibrationally excited states of both the ^35^Cl and ^37^Cl isotopologues that will be the subject of future work. Using the refined rotational constants and improved line frequencies presented here, we search for 2-chloroethanol toward 30 star-forming regions, including the protostellar binary IRAS 16293-2422 where CH_3_Cl was detected. While no detection can be confirmed, upper limits are provided, and the astrochemical implications are discussed.

## Spectral Acquisition

The millimeter spectrum was collected at the University of Wisconsin-Madison, and the spectrometer has been described previously.^19,20^ In brief, the spectrometer is composed of a microwave synthesizer (Agilent Technologies, E8257D PSG), Virginia Diodes Inc. frequency multipliers with varying doublers and triplers, an InSb Hot electron bolometer (QMC Ltd., model QFI/2BI), and a lock-in amplifier (Stanford Research Systems SR830 DSP). The spectrum was collected from 140–700 GHz at a step size of 0.1 MHz. A commercial sample of 2-chloroethanol (99%) was introduced into a 2-m long stainless-steel cell equipped with a liquid nitrogen trap and roughing pump. The base pressure of the cell was 15 mTorr, and sample pressures between 40 and 100 mTorr were used at room temperature. The microwave spectrum was collected at New College of Florida, from 8–26 GHz with 100 million averages, using a chirped-pulse Fourier transform microwave (CP-FTMW) spectrometer, described previously,^21,22^ The sample was held at room temperature and a pressure of 7 mTorr. The far-infrared spectrum was first obtained using the Australian synchrotron, explained elsewhere.^11,18^ Additional measurements performed at the Canadian Light Source (CLS) and presented here were performed using the Bruker IFS 125 HR FTIR at the full resolution of 0.00096 cm^–1^. Frequencies from about 100–600 cm^–1^ were accessed using a 6 μm Mylar beamsplitter and QMC Superconducting Niobium TES Bolometer. The spectra were recorded using a White multipass cell with a 72-m path length, equipped with polypropylene windows. The sample spectra were collected at a static sample pressure of 0.1488 Torr and averaged 205 times for optimal signal-to-noise ratio of the ν_21_ fundamental band. A nominal measurement uncertainty of 0.050 MHz was used for the rotational spectra, which is reasonable with line widths ranging from 0.8 MHz to over 2 MHz across the frequency range and the 0.100 MHz step size. A nominal measurement uncertainty of 0.000 24 cm^–1^ was used for the nominal uncertainty of the high-resolution infrared spectra.

## Computational Methods

Geometry optimization and anharmonic second-order vibrational perturbation theory (VPT2) calculations were carried out using a developmental version of CFOUR.^23^ Coupled-cluster calculations with singles, doubles, and perturbative triples, CCSD­(T), were performed with the cc-pCVTZ basis set, including the correlation of all electrons. These calculations provided vibration-rotation interaction correction constants (α values) and centrifugal distortion constants through the sextic level for both isotopologues. Additional optimizations and subsequent NBO 7.0 ^24^ calculations using Gaussian 16 ^25^ and the WebMO interface^26^ were performed at the B3LYP/6–311+G­(2d,p) level of theory to investigate the stabilization of the *Gg*′ conformer of 2-chloroethanol. Analogous calculations were performed for the 2-fluoroethanol *Gg*′ conformer for comparison.

## Results and Discussion

### Conformational Analysis

The *Gg*′ conformers of both 2-chloroethanol and 2-fluoroethanol^27–30^ are remarkably similar. The 2^nd^-order perturbation analysis of NBO 7 on the B3LYP/6–311+G­(2d,p) optimized structures of 2-chloroethanol and 2-fluoroethanol reveals an overlap allowing a weak donor–acceptor interaction, n_X_ → σ_O–H_* (Figure 2). The stabilization of the interaction is below the 2^nd^-order perturbation theory printout threshold. This observation suggests that the stabilization of the hydrogen-bond-like interactions that exist between the halogen atom and hydroxyl group is primarily electrostatic. These conformations are additionally stabilized by the *gauche* effect^30^ as evidenced by significant σ_C–H_ → σ_C–O_* and σ_C–H_ → σ_C–Cl_* interactions of 4.0 and 5.9 kcal/mol for 2-chloroethanol (4.1 and 5.0 kcal/mol for 2-fluoroethanol), respectively. The hydrogen-bond-like interaction and the *gauche* effect compensate for the less favorable steric interactions of the *Gg*′ conformer, making it the most stable conformer for both of these molecules, as well as 2-bromoethanol and 2-iodoethanol.^30^

**Figure 2:**
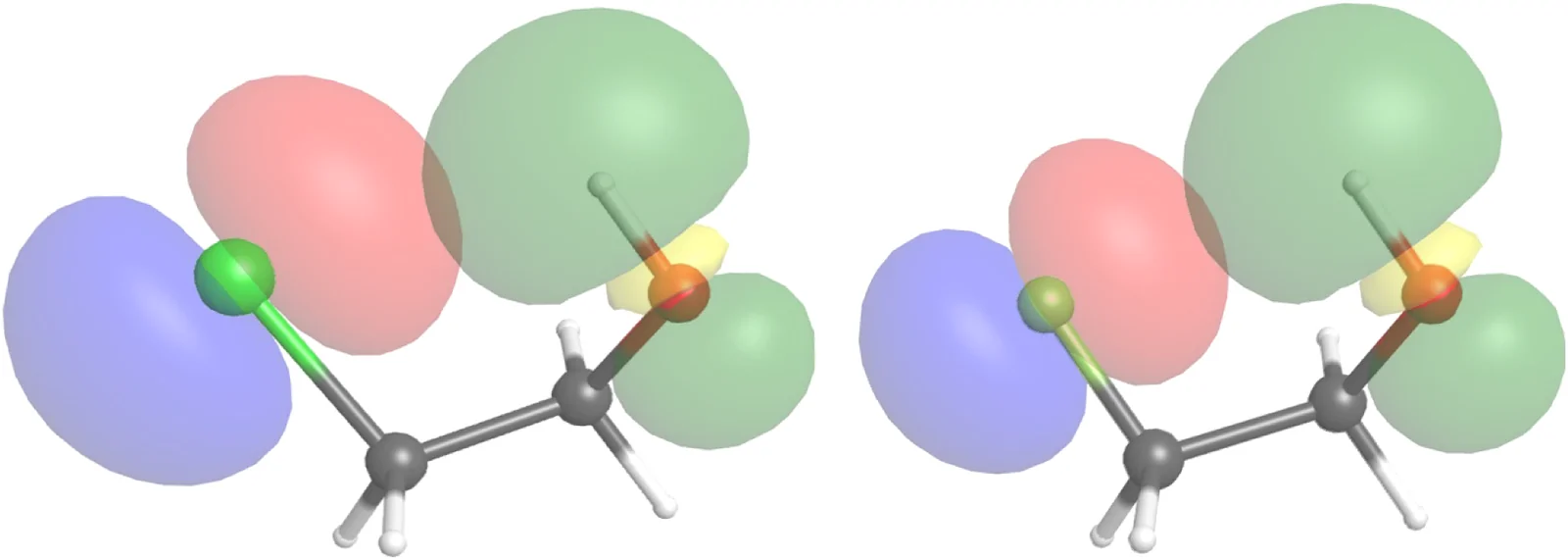
Small donor–acceptor interaction between n_X_ (red/blue, left) and σ_O–H_* (yellow/green, right) determined by NBO 7 at the B3LYP/6–311+G­(2d,p) stabilizing the *Gg*′ conformers of 2-chloroethanol (left) and 2-fluoroethanol (right).

### Ground-State Spectra for the ^35^Cl and ^37^Cl Isotopologues of 2-Chloroethanol

The *Gg′* conformer of 2-chloroethanol is a near-prolate (*κ* = −0.893) asymmetric-top with *C*_1_ symmetry and a dominant *b*-type dipole component (*μ*_*a*_ = 0.04 D, *μ*_*b*_ = 1.46 D, *μ*_*c*_ = 0.32 D, CCSD­(T)), resulting in prominent *b*-type transitions in the millimeter-wave spectrum.^11,16^ Unsurprisingly, given the spectral density of 2-chloroethanol across the room temperature rotational spectrum, the predicted relative energies of the 2-chloroethanol conformations, and their expected dipole moments, no transitions were assignable to conformers other than the lowest-energy *Gg*′ conformer. Strong ^*b*^R_1,1_ and ^*b*^R_–1,1_ transitions are observed with the selection rules Δ*K*_*a*_ = odd and Δ*K*_*c*_ = odd, as well as regular ^*b*^Q_1,–1_-branch bands separated by approximately 2*A*_0_–*B*_0_–*C*_0_ whose *K*_a_ increase with increasing frequency (Figure 3b). The most obvious features in the observed frequency range are the repeating ^*b*^Q_1,–1_ bands, which begin at *J*″ = *K*_*a*_″+1 and progress to higher frequency with increasing *J*″ and *K*_*c*_″ until a band head where the transitions then progress toward lower frequency with increasing *J*″ and *K*_*c*_″ (Figure 3a). The most intense transitions are the ^*b*^R_1,1_ and ^*b*^R_–1,1_ transitions, which form widely spaced bands of transitions that start with a *K*_*a*_ = 0 transition and progress to higher frequency with decreasing *J*″ value.

**Figure 3:**
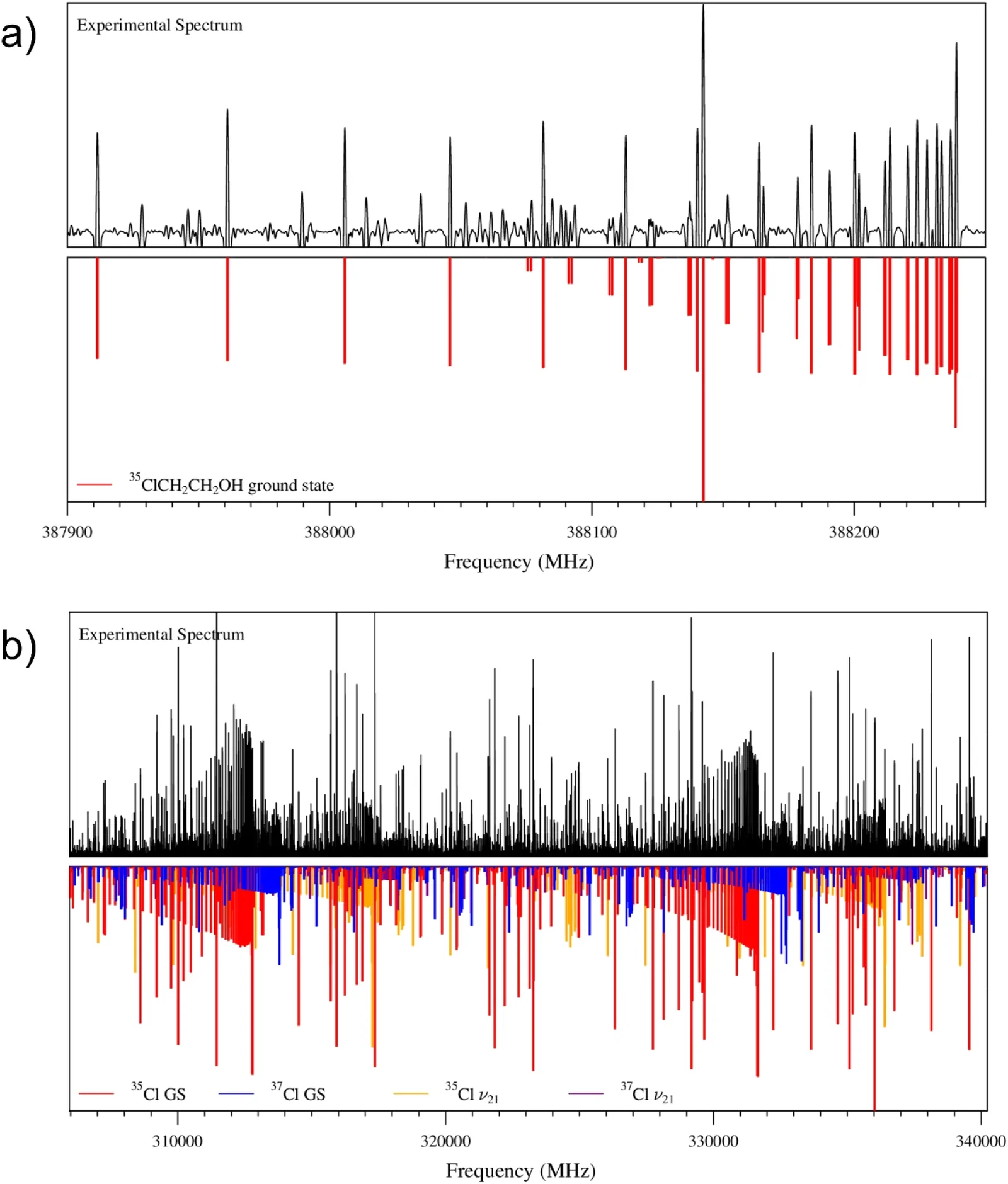
a) Example of ^35^Cl ground state Q-branch (*K_a_* ″ = 20 ^*b*^Q_1,–1_) from 387.90 to 288.25 GHz and b) spectrum from ∼305.9 to 340.2 GHz, showing the prominent ^*b*^Q_1,–1_ branch bands for ground-state (GS) and first vibrationally excited state (ν_21_) 2-chloroethanol, ^35^Cl and ^37^Cl isotopologues. Experimental spectrum (top, black) and predicted spectrum (bottom, colored) in both panels.

The ground-state constants resulting from the combined fit of the far-infrared (simultaneous fit for GS with ν_19_) and microwave spectrum^11^ were used along with the quadrupole coupling constants from the previous microwave study^16^ to initially predict the ground-state rotational spectrum of the most abundant (^35^Cl) isotopologue. The transitions were measured, assigned, and least-squares fit using an octic centrifugally distorted, A-reduced Hamiltonian in the I^*r*^ representation using the SPFIT/SPCAT program suite of Pickett^31^ and Kisiel’s Assignment and Analysis of Broadband Spectra (AABS) software package.^32,33^ A total of 5292 rotational transitions, including up to *J*″ =113 and *K*_a_″ = 37 were included in the fit (Figure 4), resulting in a root mean square (RMS) value (σ_fit_) of 0.057 MHz. The large frequency range, range of *J*″ and *K*_a_″ values of the transition energy levels, and number of transitions allowed for sufficient determination of the spectroscopic constants through the octic level of centrifugal distortion, and all transitions across this frequency range could be predicted within their nominal experimental uncertainty.

**Figure 4:**
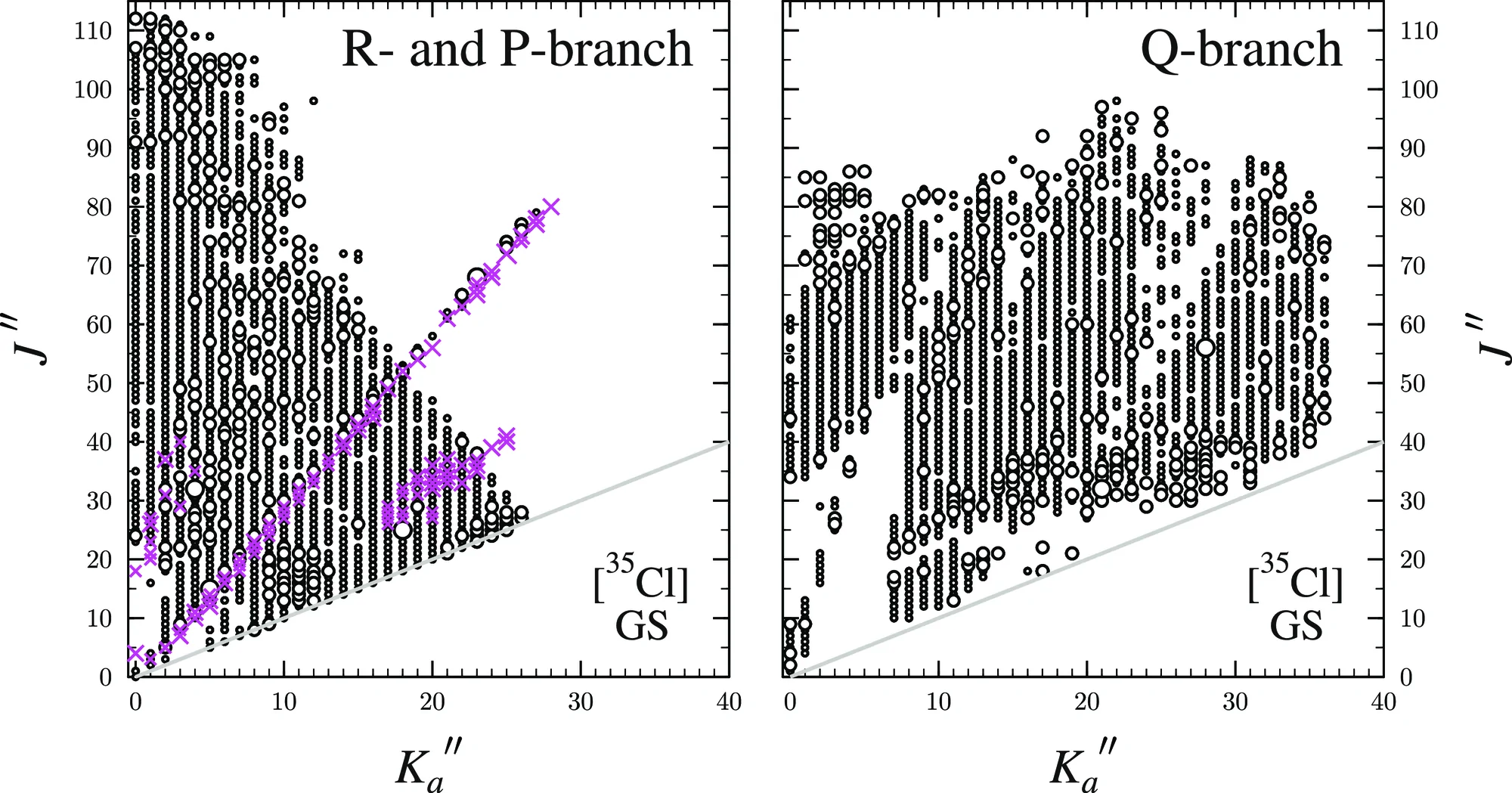
Data distribution plot for the rotational transitions of ^35^Cl isotopologue of 2-chloroethanol in the ground vibrational state (GS) from the present work. Black circles indicate R- or Q-branch transitions within their respective plots and magenta × markers indicate P-branch transitions. The size of the marker is proportional to the value of |(*f_obs_*. – *f*_*calc*._)/δ*f*|, where δ*f* is the frequency measurement uncertainty (0.1 MHz), and all values represented are smaller than 3.

The *I* = 3/2 nuclear spin of chlorine results in quadrupole coupling and splitting of rotational energy levels. We see evidence of this splitting consistently throughout the spectrum, and it is particularly prominent in the Q-branch transitions at low *J* and *K*_a_. Despite the large number of assignments and breadth of quantum numbers, the 0.057 MHz σ_fit_ value was larger than expected when compared to the results presented in previous works from this instrument, *i*.*e*., 1*H*-1,2,4-triazole,^19^ which had σ_fit_ ∼ 0.03 MHz. The larger uncertainty for 2-chloroethanol is, therefore, not expected to arise from the instrument. In order to determine if this arose from the lack of microwave spectra with well-resolved quadrupole components, new microwave spectra from 8–26 GHz were acquired. Inclusion of the 523 newly-measured microwave transitions in the fit allowed for the determination and improvement of the off-diagonal quadrupole coupling constants. Despite this improvement, the addition of these lines did not significantly lower the overall σ_fit_ value, which remained 0.057 MHz. The previous microwave analysis of 2-chloroethanol^15,16^ did not report hyperfine-resolved transition frequencies, but rather the splitting. As the microwave spectrum was remeasured and extended in the work reported here, this previous work was not included in the presented fit. The spectrum of 2-chloroethanol at room temperature is very dense (Figure 3) due to the many low-lying vibrationally excited states from both chlorine isotopologues (*vide infra*) and the relatively small value of 2*C*_0_, which controls the R-branch band separation. As a result of the spectral density, the observed transition frequencies may be slightly skewed due to underlying transitions reducing the ability to accurately measure the transition line centers. The poorly measured line centers would slightly increase the uncertainty in the least-squares fitting. Assessing the quality of the spectroscopic constants further and exploring the source of the larger than expected σ_fit_ value would be facilitated by obtaining computed octic centrifugal distortion constants for 2-chloroethanol, which are currently beyond the capabilities of commonly available computational software.

The spectroscopic constants obtained for the ^35^Cl ground state, modeled with an octic centrifugally distorted rotor Hamiltonian including nuclear quadrupole coupling, are presented in Table 1, compared to the CCSD­(T) computed values. The *A*_0_, *B*_0_, and *C*_0_ rotational constants from the combined IR+MW fit reported by Soliday *et al.* ^11^ and the computed rotational constants vary by ≤0.0004% and ≤0.7%, respectively, from the newly determined values presented in this work. The computed values of quartic centrifugal distortion constants are all within 7% and the previous experimental values of IR+MW fit^11^ are within 1% of their corresponding values determined in this work. The computed sextic centrifugal distortion values are within 20% of the experimentally determined values. While there is exceptionally close agreement between combined IR+MW fit^11^ and the present work for the rotational and quartic distortion constants, the sextic constants agree only to within about 35%. Inclusion of the additional sextic and octic terms reported here has, therefore, clearly resulted in a more reliable determination of *Φ*_*JK*_, *Φ*_*KJ*_, and *Φ*_*K*_ than that from the previous literature, particularly with improved agreement of *Φ*_*JK*_. The strong agreement between experimentally and computationally determined quartic and sextic values shown here provides confidence in the CCSD­(T) predicted values. Therefore, inclusion of computed terms where they could not be experimentally determined, instead of holding these values constant at zero, would have likely improved this agreement in the previous work. Current computational software packages do not predict octic centrifugal distortion constants, however, their inclusion in the fit presented here was necessary for the accurate determination of the lower-order centrifugal distortion constants. The inclusion of well-resolved quadrupole split transitions from the microwave spectra collected and analyzed in this work has resulted in the determination of all on- and off-diagonal quadrupole coupling terms. Each quadrupole coupling term is in agreement with the previous microwave work, within the corresponding statistical uncertainty and significant improvement is observed for all constants, particularly *χ*_*ab*_. It is important to note that because of the higher σ_fit_ value in this work, compared to previous studies with this instrumentation, the use of the catalog provided here for frequencies beyond what is measured should be conducted with care.

**Table 1:** Spectroscopic Constants for the Ground Vibrational State of the ^35^Cl and ^37^Cl Isotopologues of 2-Chloroethanol (A-Reduced Hamiltonian and I*^r^* Representation)

	^35^Cl			^37^Cl		
	Previous works^a^	CCSD(T)^b^	Experimental	Previous works^a^	CCSD(T)^b^	Experimental
*A*_0_ (MHz)	12747.9598 (23)	12717	12747.961 93 (20)	12705.922 3 (47)	12705	12705.917 73 (17)
*B*_0_ (MHz)	3505.79892 (68)	3480	3505.789 127 (62)	3423.540 2 (16)	3399	3423.536 116 (58)
*C*_0_ (MHz)	2981.26762 (44)	2961	2981.257 712 (49)	2919.498 0 (10)	2901	2919.494 355 (49)
*Δ*_*J*_ (kHz)	2.973 30 (23)	2.92	2.963 697 (22)	2.84605 (75)	2.80	2.839 395 (22)
*Δ*_*JK*_ (kHz)	–14.569 7 (40)	–13.8	–14.565 22 (18)	–14.026 (14)	–13.4	–14.042 96 (17)
*Δ*_*K*_ (kHz)	50.925 6 (81)	47.6	50.923 44 (41)	50.237 (31)	47.3	50.164 11 (42)
*δ*_*J*_ (kHz)	0.737 69 (10)	0.718	0.736 653 8 (69)	0.696 32 (39)	0.677	0.694 279 8 (71)
*δ*_*K*_ (kHz)	7.572 (12)	7.27	7.629 68 (32)	7.352 (45)	7.12	7.449 83 (33)
*Φ*_*J*_ (Hz)		–0.003 29	–0.002 864 2 (40)		–0.003 05	–0.002 663 0 (36)
*Φ*_*JK*_ (Hz)	0.062 4 (10)	0.089 1	0.092 454 (85)	0.082 6 (56)	0.084 9	0.087 00 (10)
*Φ*_*KJ*_ (Hz)	–0.571 2 (42)	–0.623	–0.678 55 (41)	–0.630 (21)	–0.605	–0.650 76 (34)
*Φ*_*K*_ (Hz)	1.455 6 (54)	1.41	1.592 49 (60)	1.587 (26)	1.40	1.556 72 (55)
*ϕ*_*J*_ (Hz)		–0.001 00	–0.000 8511 (17)		–0.000 904	–0.000 788 6 (13)
*ϕ*_*JK*_ (Hz)		–0.043 1	–0.045 26 (12)		–0.041 0	–0.042 895 (88)
*ϕ*_*K*_ (Hz)		0.275	0.324 3 (23)		0.282	0.315 6 (22)
*L*_*J*_ (mHz)			0.000 020 45 (30)			0.000 018 94 (12)
*L*_*JJK*_ (mHz)			0.000 334 (13)			0.000 366 4 (73)
*L*_*JK*_ (mHz)			–0.005 87 (30)			–0.005 684 (33)
*L*_*KKJ*_ (mHz)			0.032 65 (98)			0.031 26 (10)
*L*_*K*_ (mHz)			–0.067 08 (76)			–0.065 99 (23)
*l*_*J*_ (mHz)			0.000 008 98 (14)			[0.000 008 98]^c^
*l*_*JK*_ (mHz)			–0.000 126 (11)			[−0.000 126]^c^
*l*_*KJ*_ (mHz)			0.008 03 (37)			[0.008 03]^c^
*l*_*K*_ (mHz)			0.024 4 (54)			[0.024 4]^c^
*χ*_*aa*_ (MHz)	–19.16 (10)		–19.107 (30)		–15.843 (55)	–15.661 (55)
*χ*_*bb*_ – χ_*cc*_ (MHz)	–44.47 (14)		–44.471 (25)		–34.69 (14)	–34.620 (48)
*χ*_*ab*_ (MHz)	50 (2)		49.33 (16)			
*χ*_*bc*_ (MHz)	–14.7 (3)		–14.84 (60)			
*χ*_*ac*_ (MHz)	14.7 (3)		14.833 (81)			
*N*_*lines*_ ^d^			5292			2882
*σ*_*fit*_ (MHz)			0.057			0.038

a Rotational and centrifugal distortion constants from Soliday et al.^11^ and the nuclear quadrupole coupling constants are from Azrak and Wilson.^16^

b Evaluated with the cc-pCVTZ basis set.

c Values in brackets are held constant at the corresponding ^35^Cl values.

d Independent frequencies.

The less-abundant, ^37^Cl, isotopologue is also observed in the spectrum at the expected intensity ratio of 3:1 compared to transitions corresponding to the ^35^Cl isotopologue (Figure 2). Similar spectral features to those of the normal isotopologue are observed. We have assigned 2885 independent rotational transitions, resulting in the spectroscopic constants presented in Table 1. The ground vibrational state ^37^Cl data distribution plot is available in Figure S3. Similar agreement is observed between the computed and previous literature values for the spectroscopic constants of the ^37^Cl isotopologue and the experimentally determined values presented here. The largest discrepancies between the computed and currently determined values are for *Φ*_*J*_ and *ϕ*_*J*_, which agree within 27% as opposed to the 13% for all other values. This is likely due to their smaller magnitude. All on-diagonal octic centrifugal distortion constants are determined reasonably well, with errors at least an order of magnitude smaller than their value. The off-diagonal parameters, however, could not be determined and are held constant at the ^35^Cl values, as computational predictions are not available for these values. Despite this limitation, the overall σ_fit_ value for the ^37^Cl isotopologue is lower (0.038 MHz) than for the ^35^Cl isotopologue. The reasons for this improvement to the least-squares fit are not entirely clear.

Similar to the ^35^Cl isotopologue, quadrupole coupling is observed and is particularly prominent in the Q-branch regions with larger separation at low *J*. The on-diagonal quadrupole coupling parameters were reported in the previous microwave work.^15,16^ The additional 156 microwave spectral assignments helped to refine the quadrupole coupling constants, however, unlike for the main isotopologue, inclusion of all parameters was not possible. Therefore, we report *χ*_*aa*_ and *χ*_*bb*–*cc*_, both of which are in agreement with the literature values within the corresponding statistical uncertainties.

### Spectral Analysis of the Isolated ν_21_ Vibrationally Excited State for the ^35^Cl and ^37^Cl Isotopologues of 2-Chloroethanol

A complete set of spectroscopic constants through the octic centrifugal distortion constants has been determined for the ν_21_ state of the ^35^Cl isotopologue. Four octic terms had to be held constant for the corresponding [^37^Cl]-2-chloroethanol vibrational state. These constants are shown in Table 2 and data distribution plots for ν_21_^35^Cl and ^37^Cl are available in Figures S4–S6. For the normal isotopologue, *A*_21_, *B*_21_, and *C*_21_ rotational constants are within 0.0006% of the previously determined values and within 0.0034% for the ^37^Cl isotopologue.^15,16^ The experimental quartic distortion constants have only been previously reported for the ^35^Cl isotopologue in the ν_21_ excited state,^18^ and these values are in excellent agreement with the current work (within 0.25%). The additional sextic and octic distortion constants are all reasonably determined and comparable in magnitude and sign to the corresponding ground-state constants, indicating a physically meaningful fit. The computationally predicted vibration-rotation interaction (α) values are within 7% of their corresponding experimental values, the largest absolute discrepancy is 1.6 MHz. The close agreement between the computed and experimentally determined α terms for ν_21_ indicates that this state is isolated and the centrifugal distortion has been appropriately characterized with a single-state Hamiltonian (Table 3). Additionally, the energy of ν_21_ for [^35^Cl]-2-chloroethanol, 150.908 599 5 (43) cm^–1^, is determined for the first time using the high-resolution infrared spectra acquired in this work (Figure 5). The ν_21_ vibrational frequency of 2-fluoroethanol, 151.785 376 (13) cm^–1^, recently determined by high-resolution synchrotron far-infrared and microwave spectroscopy,^29^ is quite close to that for ν_21_ of 2-chloroethanol. As a result of the closeness of the ν_21_ vibrational frequencies and molecular structures, small changes in the quartic centrifugal and vibration-rotation interaction constants (*B*_0_ – *B*_21_) for 2-fluoroethanol and 2-chloroethanol are observed. These observed small changes in the centrifugal distortion constants are predominantly due to the changes in the rotational constants, related to the different halogen atoms.

**Figure 5:**
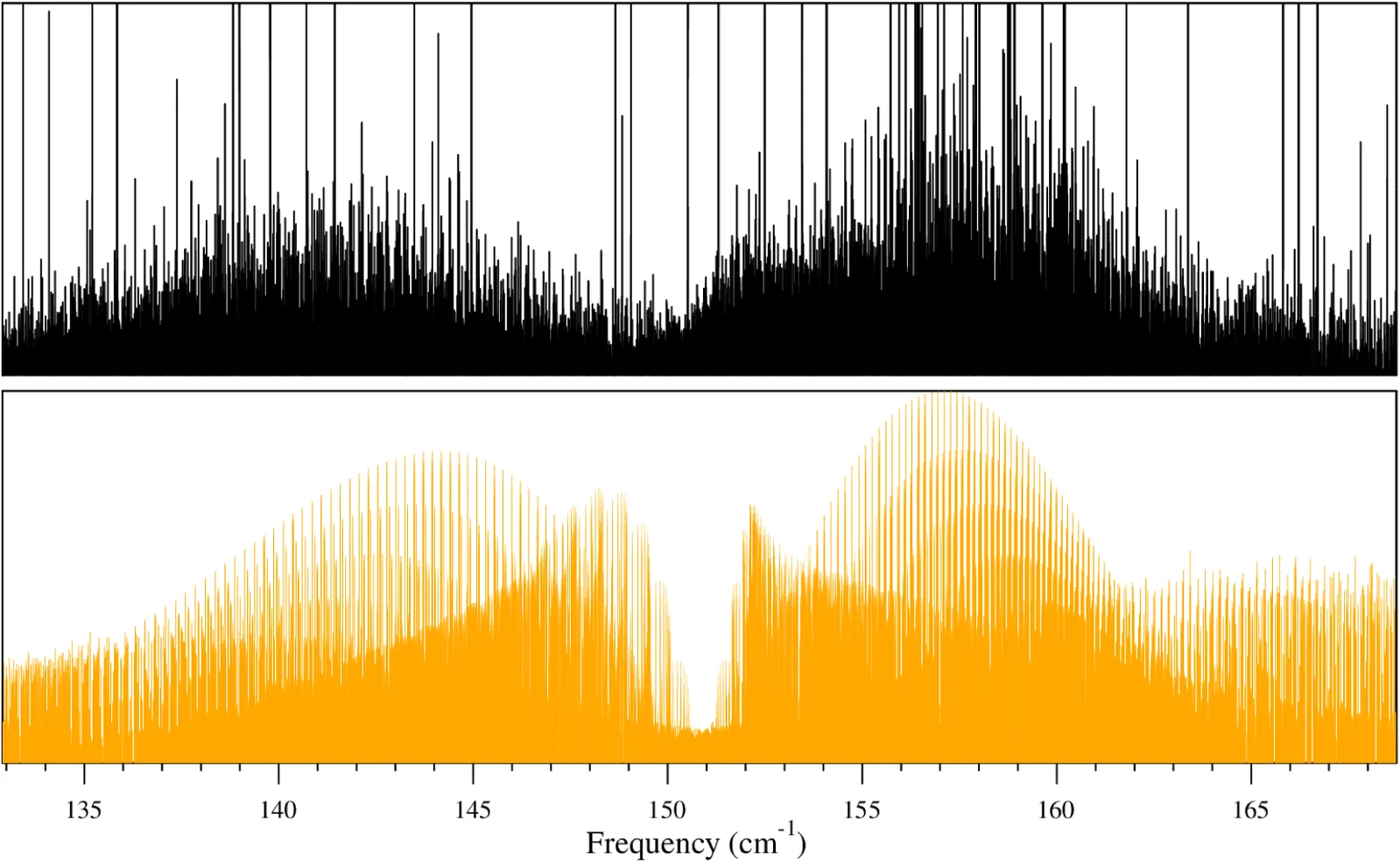
Predicted stick spectrum of ν_21_^35^Cl isotopologue of 2-chloroethanol (orange, bottom) and the experimental high-resolution infrared spectrum of 2-chloroethanol with ^37/35^Cl isotopes at natural abundance (black, top) from ∼130 to 170 cm^–1^.

**Table 2:** Spectroscopic Constants for the Lowest-Energy Vibrationally Excited State (ν_21_) of the ^35^Cl and ^37^Cl Isotopologue of 2-Chloroethanol (A-Reduced Hamiltonian and I*^r^* Representation)

	^35^Cl	^37^Cl
*A*_*21*_ (MHz)	12865.416 30 (21)	12822.275 77 (23)
*B*_*21*_ (MHz)	3480.043 114 (69)	3398.652 219 (91)
*C*_*21*_ (MHz)	2965.533 423 (54)	2904.083 377 (76)
*Δ*_*J*_ (kHz)	2.891 896 (26)	2.770 673 (36)
*Δ*_*JK*_ (kHz)	–15.164 61 (23)	–14.629 84 (25)
*Δ*_*K*_ (kHz)	56.144 44 (54)	55.297 69 (75)
*δ*_*J*_ (kHz)	0.716 604 (11)	0.675 524 (12)
*δ*_*K*_ (kHz)	7.903 90 (41)	7.716 40 (50)
*Φ*_*J*_ (Hz)	–0.002 1046 (55)	–0.001 945 6 (63)
*Φ*_*JK*_ (Hz)	0.090 32 (14)	0.086 60 (17)
*Φ*_*KJ*_ (Hz)	–0.734 67 (61)	–0.710 26 (55)
*Φ*_*K*_ (Hz)	1.861 77 (89)	1.830 8 (11)
ϕ_*J*_ (Hz)	–0.000 620 7 (27)	–0.000 563 9 (21)
ϕ_*JK*_ (Hz)	–0.048 30 (17)	–0.045 28 (13)
ϕ_*K*_ (Hz)	0.372 8 (36)	0.384 9 (36)
*L*_*J*_ (mHz)	0.000 0159 3 (44)	0.000 014 89 (28)
*L*_*JJK*_ (mHz)	0.000 410 (23)	0.000 372 (11)
*L*_*JK*_ (mHz)	–0.004 10 (58)	–0.004 184 (49)
*L*_*KKJ*_ (mHz)	0.029 9 (18)	0.030 64 (17)
*L*_*K*_ (mHz)	–0.079 0 (14)	–0.083 08 (54)
*l*_*J*_ (mHz)	0.000 006 91 (22)	[0.000 006 91]^a^
*l*_*JK*_ (mHz)	–0.000 112 (16)	[−0.000 112]^a^
*l*_*KJ*_ (mHz)	0.010 29 (63)	[0.010 28]^a^
*l*_*K*_ (mHz)	0.064 (10)	[0.064]^a^,^b^
χ_*aa*_ (MHz)	–19.615 (51)	–16.33 (11)
χ_*bb*_ – χ_*cc*_ (MHz)	–43.740 (35)	–34.020 (72)
χ_*ab*_ (MHz)	50.34 (49)	
χ_*bc*_ (MHz)	–8.71 (53)	
χ_*ac*_ (MHz)	5.51 (53)	
*E*_21_ (cm^–1^)	150.908 660 0 (43)	
*N*_lines_ ^b^	3788/936	2110
*σ*_fit_ (MHz)^c^	0.049/4.2	0.043

a Values in brackets are held constant at the corresponding ^35^Cl values.

b Independent frequencies of the rotational/high-resolution infrared transitions.

c *σ*_fit_ values from the fitting of rotational/high-resolution infrared transitions.

**Table 3:** Experimental and Computed Vibration-Rotation Interaction Constants for Excited Vibrational States of 2-Chloroethanol

2-Chloroethanol	*B*_0_ *– B*_*v*_	Experimental	CCSD(T)	obs. – calc.
[^35^Cl]-	*A*_0_ *– A*_21_ (MHz)	–117.454 37 (29)	–116.36	–1.09
	*B*_0_ *– B*_21_ (MHz)	25.746 014 (94)	27.35	–1.60
	*C*_0_ *– C*_21_ (MHz)	15.724 288 (73)	16.75	–1.03
[^37^Cl]-	*A*_0_ *– A*_21_ (MHz)	–116.358 04 (29)	–115.96	–0.40
	*B*_0_ *– B*_21_ (MHz)	24.883 89 (11)	26.45	–1.57
	*C*_0_ *– C*_21_ (MHz)	15.410 971 (90)	16.44	–1.03

Additional vibrational modes up to 500 cm^–1^ (Figure S2) have been identified. The second lowest-energy fundamental (ν_20_) and first overtone (2ν_21_) excited states are strongly Coriolis-coupled, and the assignment of higher *J* and *K*_a_ transitions of ν_19_ (the next-higher excited state in energy order) shows weak coupling to 2ν_21_, thereby creating a Coriolis-coupled triad. The interactions between these three states are still under investigation and will be the focus of a follow-up manuscript. The additional four vibrationally excited states including the fourth lowest-energy fundamental, ν_18_, the second overtone, 3ν_21_, and two combination bands, ν_21+20_ and ν_21+19_, have also been observed as a complex Coriolis-coupled polyad.

### Astronomical Implications

Using the catalog produced here, which provides refined rotational constants and improved transition frequencies, 2-chloroethanol was sought toward the protostar IRAS 16293-2422 B, the only currently known location where an organohalogen, CH_3_Cl, has been detected, along with 29 additional regions of the Galactic interstellar medium characterized by chemical complexity, including low- and high-mass star forming regions, protostellar outflows, HII regions and others. The details of this search are presented in Supporting Information. The column density upper limit in IRAS 16293-2422 B of 4 × 10^14^ cm^–2^ is comparable to the observed CH_3_^35^Cl column density of (4.6 ± 0.7) × 10^14^ cm^–2^ in this region.^4^ Due to the increased complexity and therefore partition function from CH_3_^35^Cl to HOCH_2_CH_2_Cl, this upper limit is not particularly constraining. The column density upper limits in the 29 additional sources range from 1.6 × 10^11^ to 4.2 × 10^15^ cm^–2^. In order to provide more impactful conclusions from these upper limits, additional chlorine chemistry needs to be investigated in these sources.

## Conclusions

The millimeter/submillimeter spectrum of 2-chloroethanol from 140–700 GHz is reported here with direct comparison to radio astronomical data. The analysis of the ground state and first vibrationally excited state of both ^35^Cl and ^37^Cl isotopologues has provided refined rotational constants and centrifugal distortion constants up to and including the octic level. The inclusion of new microwave data with well-resolved quadrupole splitting has resulted in the refinement of the quadrupole coupling constants, with particular improvement for the off-diagonal terms. Additionally, we provide the first accurate and precise energy determination for ν_21_ for [^35^Cl]-2-chloroethanol, 150.908 599 5 (43) cm^–1^.

The density of the millimeter-wave spectrum of 2-chloroethanol results in underlying vibrational structure that causes a slightly larger uncertainty in the final fit when compared to previous spectral fits using the same instrumentation.^19,20,34^ Therefore, the fit presented here for 2-chloroethanol does not extrapolate well outside of the frequency region collected. We have, however, provided not only accurate spectral constants that match the spectrum from 140–700 GHz, but also a line list in a frequency range that can be directly compared to data from radio telescopes. Therefore, the spectral information presented here for the ground state dominant (^35^Cl) isotopologue of 2-chloroethanol is essential to its identification in the interstellar medium. The accurate spectroscopic catalog and corresponding partition functions provided here for 2-chloroethanol indicate that its continued interstellar elusivity, even in the only location of known organohalogen chemistry (IRAS16293), is due to its low abundance combined with its large partition function. This is not particularly surprising given the overall interstellar abundance of chlorine.

## Supplementary Material





## Data Availability

The data that support the findings of this study are available within the article and its Supplementary Material.

## Notes

Details of the Astronomical Search and data distribution plots for all isotopologues (PDF)
Least-squares fitting for all isotopologues and and computational output files (ZIP)

## ⟁

Max Planck Institute for Extraterrestrial Physics, Garching, 85748, Germany
