# Supplementary material for: A High-Resolution Rotational Analysis of 2‑Chloroethanol (HOCH2CH2Cl) and Its First Vibrationally Excited State
Source: ACS Earth Space Chem. 2026 Sep 21;10(10):2402–10. doi: 10.1021/acsearthspacechem.6c00221 (PMC13679090; doi:10.1021/acsearthspacechem.6c00221)
Supplement: Supplementary file 1 [file sp6c00221_si_001.pdf]

A High-Resolution Rotational Analysis of 2-Chloroethanol ($HOCH_2CH_2Cl$) and its First Vibrationally Excited State – Supporting Information

Hayley A. Bunn,[1,2] *Holdson H. Liang,*[1] *Brian J. Esselman,*[1] *Paul L. Raston,*[3] *Andi Pracko,*[4]

Steven Shipman,[5] *Brant Billinghurst,*[6] *Jianbao Zhao,*[6] *Jes K. Jørgensen,*[7]

Susanna L. Widicus Weaver [1,8*]

[1] Department of Chemistry, University of Wisconsin–Madison, Madison, Wisconsin 53706, USA
[2] Current affiliation: Max Planck Institute for Extraterrestrial Physics, Garching, 85748, Germany
[3] Department of Chemistry, University of Hawai'i at Mānoa, Honolulu, HI 96822, USA
[4] Department of Chemistry, New College of Florida, Sarasota, FL, 34243, USA
[5] BrightSpec, Inc., 770 Harris St., Suite 104b, Charlottesville, VA, USA
[6] Canadian Light Source. Inc., University of Saskatchewan, Saskatoon, Saskatchewan S7N 2 V3, Canada
[7] Niels Bohr Institute, University of Copenhagen, Jagtvej 155A, DK-2200 Copenhagen N., Denmark
[8] Department of Astronomy, University of Wisconsin–Madison, Madison, Wisconsin 53706, USA

TOC Graphic

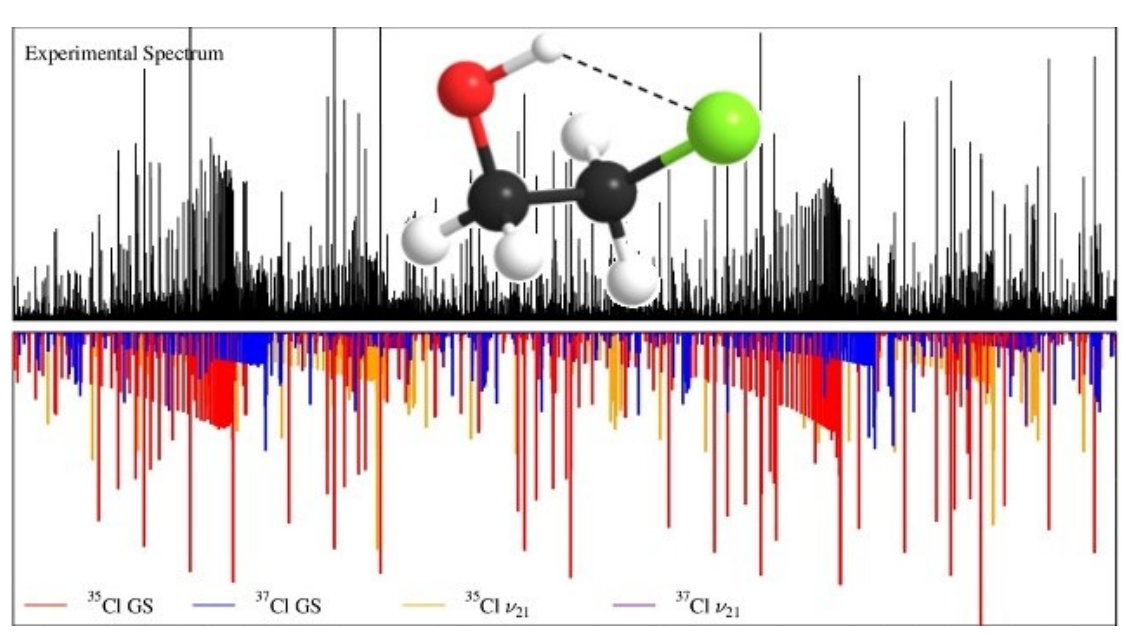

Table of Contents	Page
**Astronomical Search**	S1-S12
**Figure S1**. Search summary for strongly predicted 2-chloroethnaol lines towards IRAS 16293.	S4-S9
**Figure S**. Vibrational energy Level Diagram for 2-chloroethanol	S13
**Figure S3-S6**. Data distribution plot for the least-squares fit of millimeter-wave spectroscopic data for the normal isotopologue of oxazole	S13-S16
**References**	S17
**Least-squares fitting files (.lin, .par, .int, and .res)**	Separate files
**B3LYP/6-311+G(2d,p) opt and NBO 7 output files from Gaussian 16**	Separate files
**CCSD(T)/cc-pCVTZ anharmonic frequency output files from CFOUR**	Separate files

Astronomical search and astronomical implications

IRAS 16293

Using the catalog produced here, providing refined rotational constants and improved transition frequencies, 2-chloroethanol was sought towards the protostar IRAS 16293-2422 B, the only currently known location where an organohalogen, CH_3Cl, has been detected. We used the spectroscopic data from the Atacama Large Millimeter/Submillimeter Array Protostellar Interferometric Survey (PILS), which is an unbiased wideband survey with frequency coverage from 329.1 to 362.9 GHz with a velocity resolution of ~0.2 km/s and an angular resolution of 0"5 (70 au). We searched for the brightest predicted lines from the ground-state spectral catalog of the ^{35}Cl isotopologue of 2-chloroethanol using a temperature of 100 K, based on the excitation temperature of the observed $CH_3{}^{35}Cl$. It is not surprising that due to the density of this molecular spectrum, some transitions appear to align with those in the observation. However, there are strongly predicted lines that are not identified (see Figure S1) and therefore no detection of this species can be confirmed. A best estimate of the upper limit is derived to be $4x10^{14}$ cm^{-2}, using segments where no matches were observed. The upper limit column density reported in IRAS 16293-2422 B is comparable to the observed $CH_3{}^{35}Cl$ column density of $(4.6 \pm 0.7) \times 10^{14}$ cm^{-2} in this region. Due to the increased complexity and therefore partition function from $CH_3{}^{35}Cl$ to $HOCH_2CH_2Cl$, this upper limit is not particularly constraining.

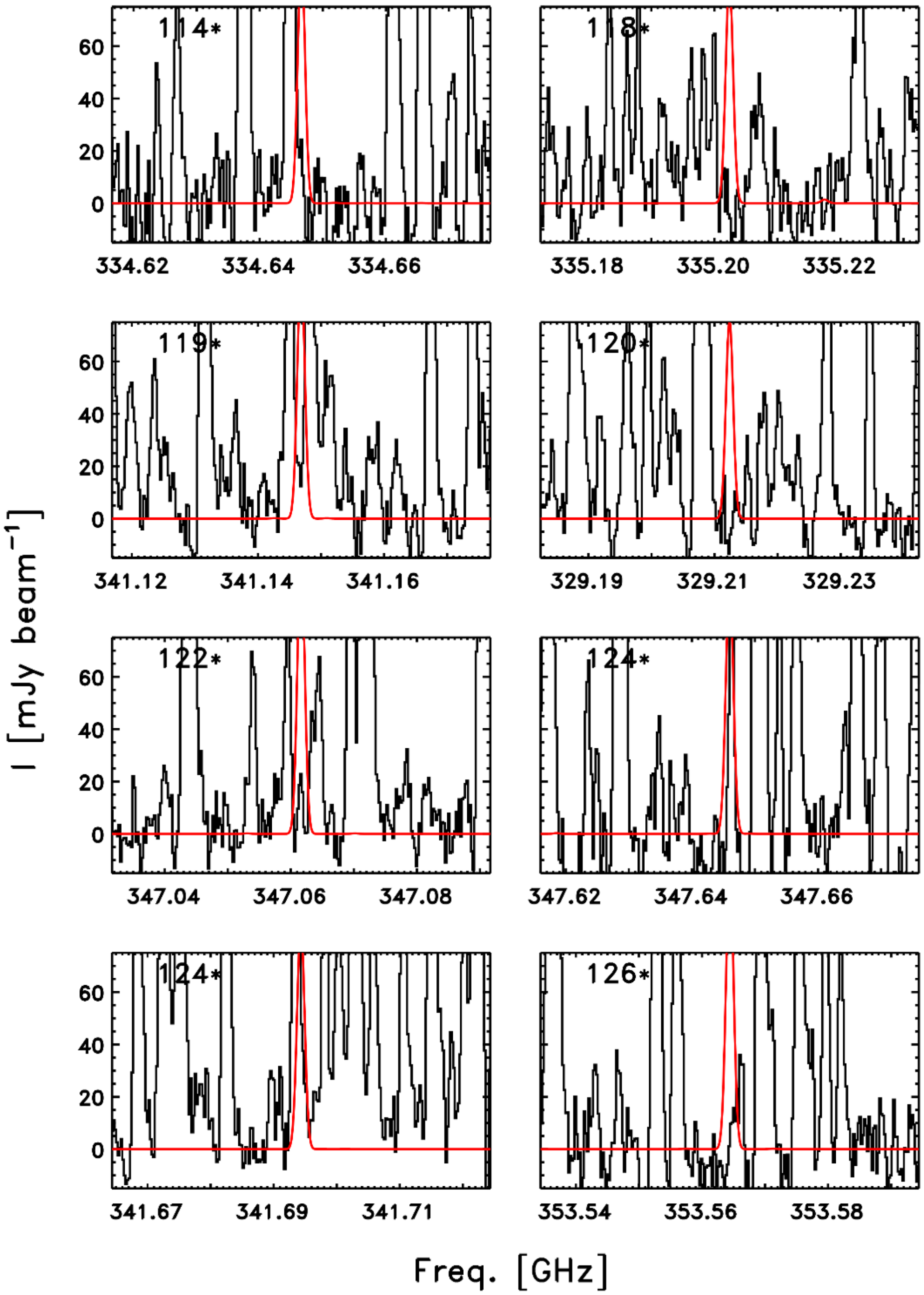

114*
118*
119*
120*
122*
124*
124*
126*
334.62
334.64
334.66
335.18
335.20
335.22
341.12
341.14
341.16
329.19
329.21
329.23
347.04
347.06
347.08
347.62
347.64
347.66
341.67
341.69
341.71
353.54
353.56
353.58
0
20
40
60
I [mJy beam^{-1}]
Freq. [GHz]

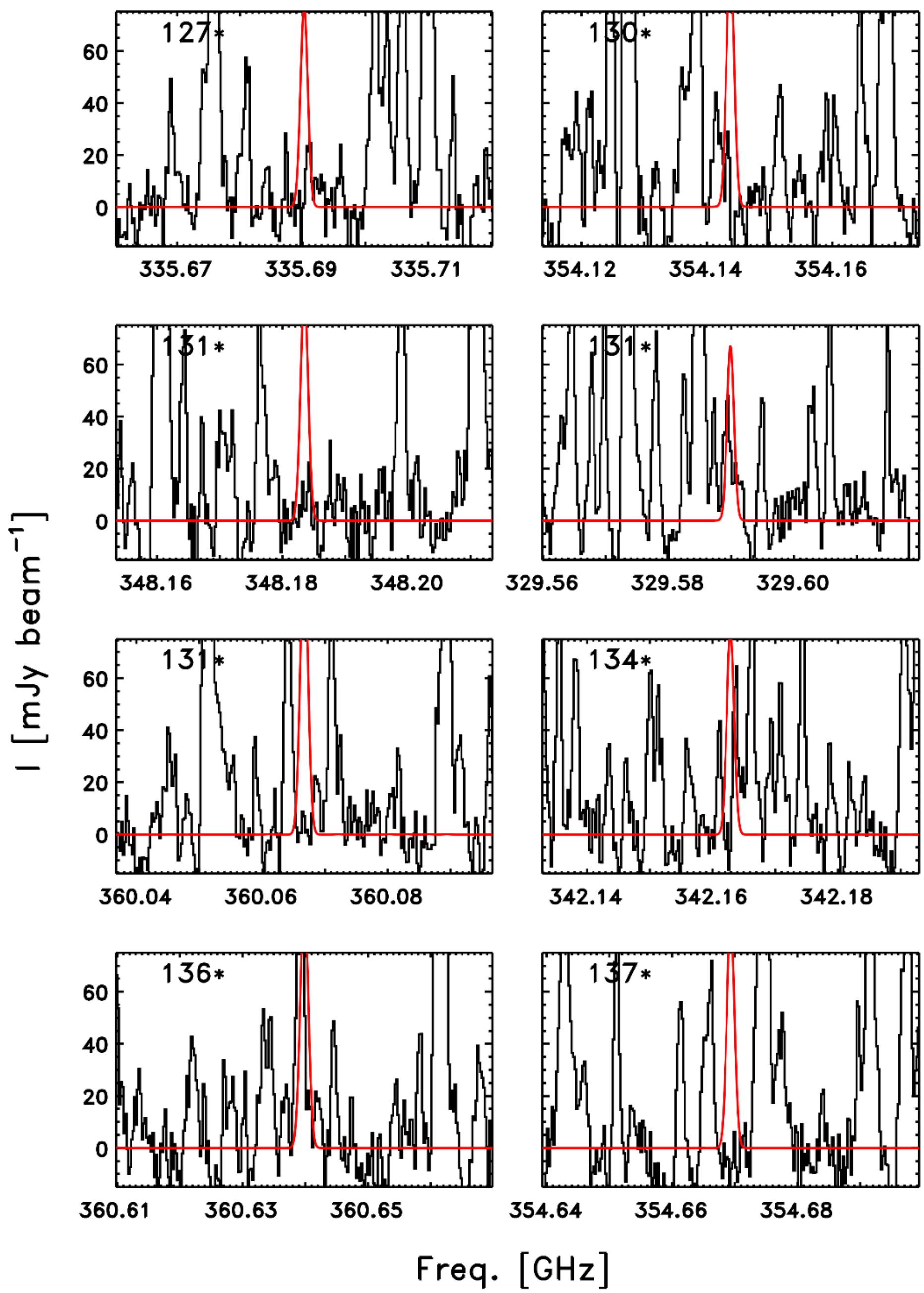
127*
130*
131*
131*
131*
134*
136*
137*
I [mJy beam^{-1}]
Freq. [GHz]
335.67 335.69 335.71
354.12 354.14 354.16
348.16 348.18 348.20
329.56 329.58 329.60
360.04 360.06 360.08
342.14 342.16 342.18
360.61 360.63 360.65
354.64 354.66 354.68
0 20 40 60

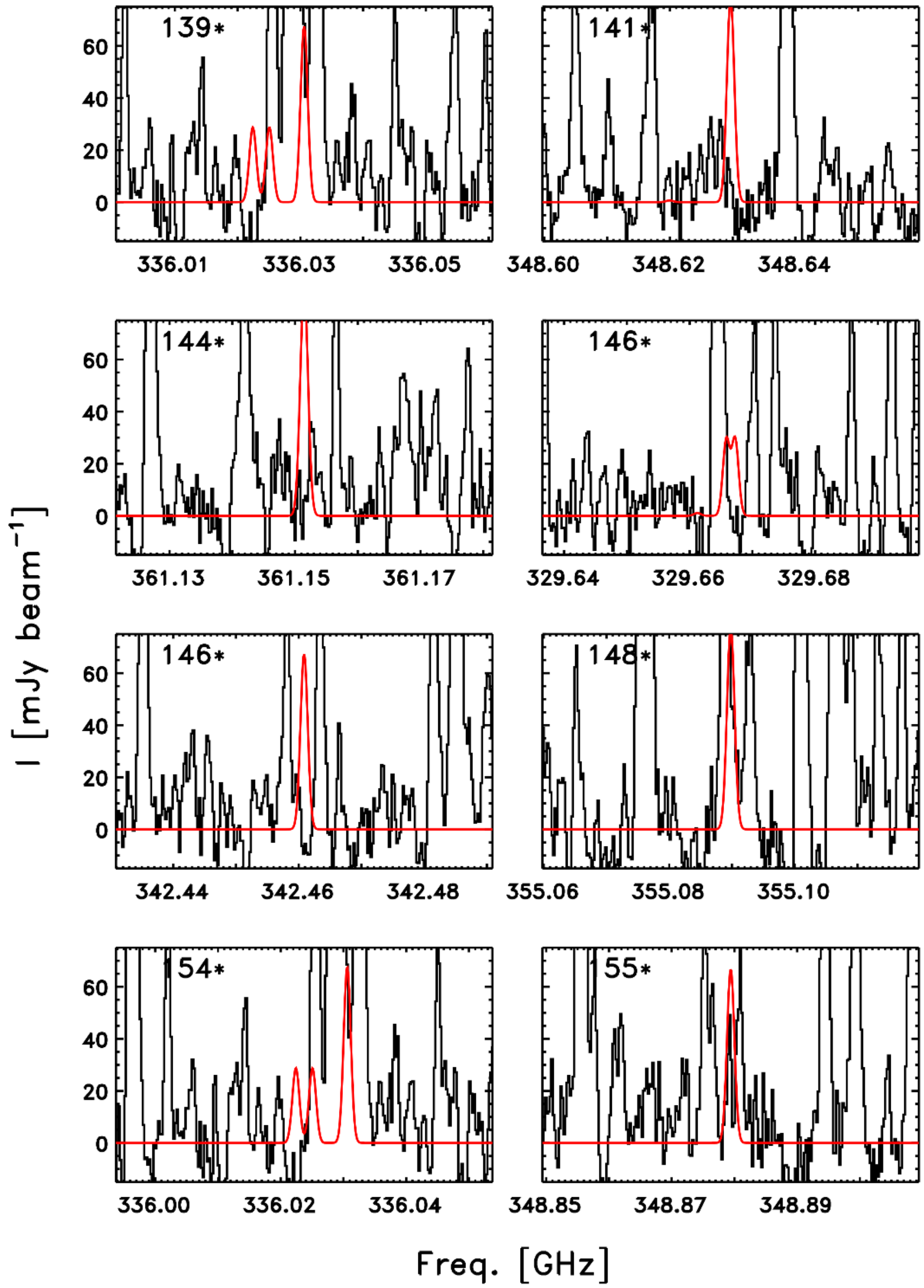

139*
141*
144*
146*
146*
148*
154*
55*
I [mJy beam^{-1}]
Freq. [GHz]

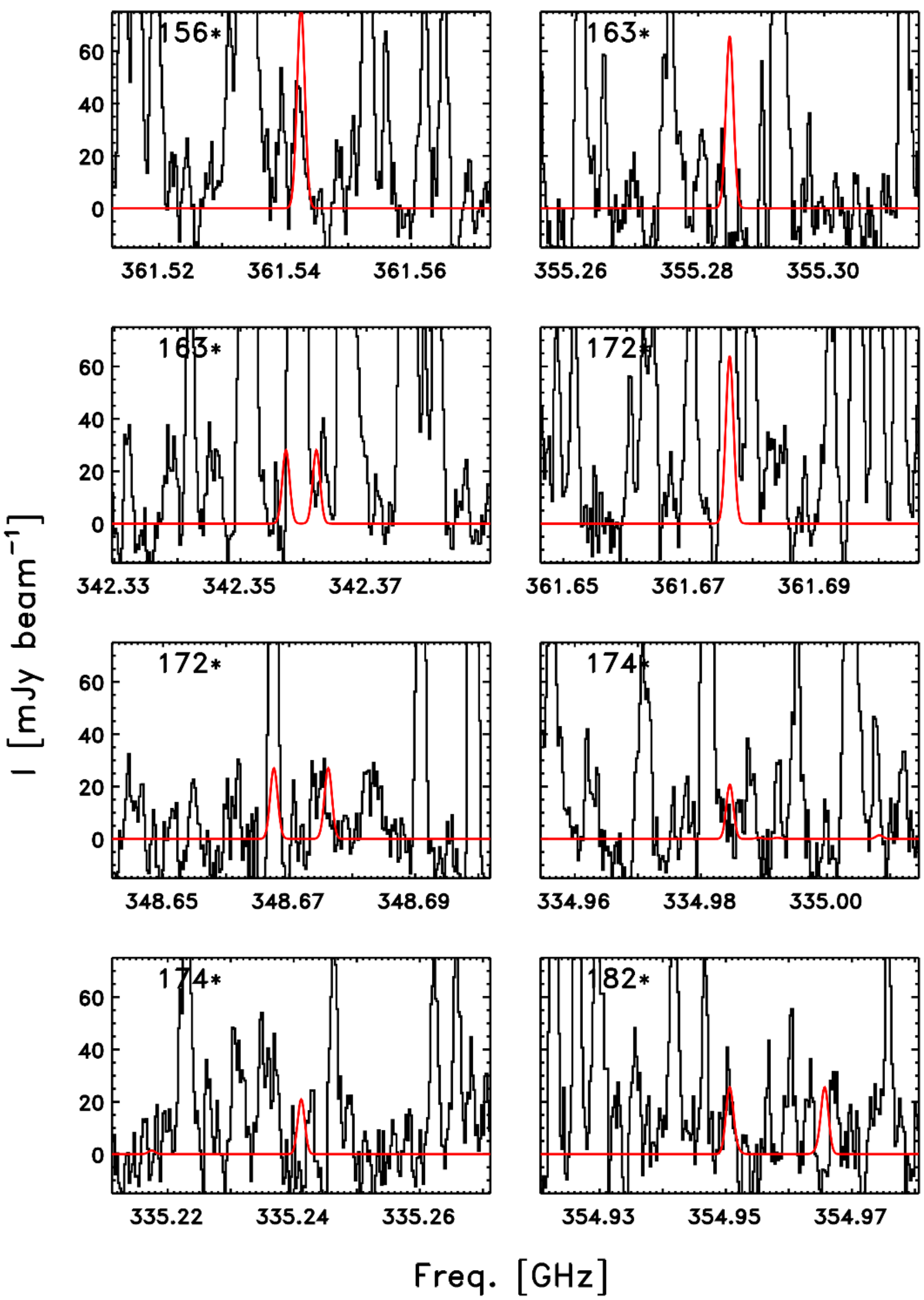
156*
163*
163*
172*
172*
174*
174*
182*
I [mJy beam^{-1}]
Freq. [GHz]
361.52
361.54
361.56
355.26
355.28
355.30
342.33
342.35
342.37
361.65
361.67
361.69
348.65
348.67
348.69
334.96
334.98
335.00
335.22
335.24
335.26
354.93
354.95
354.97

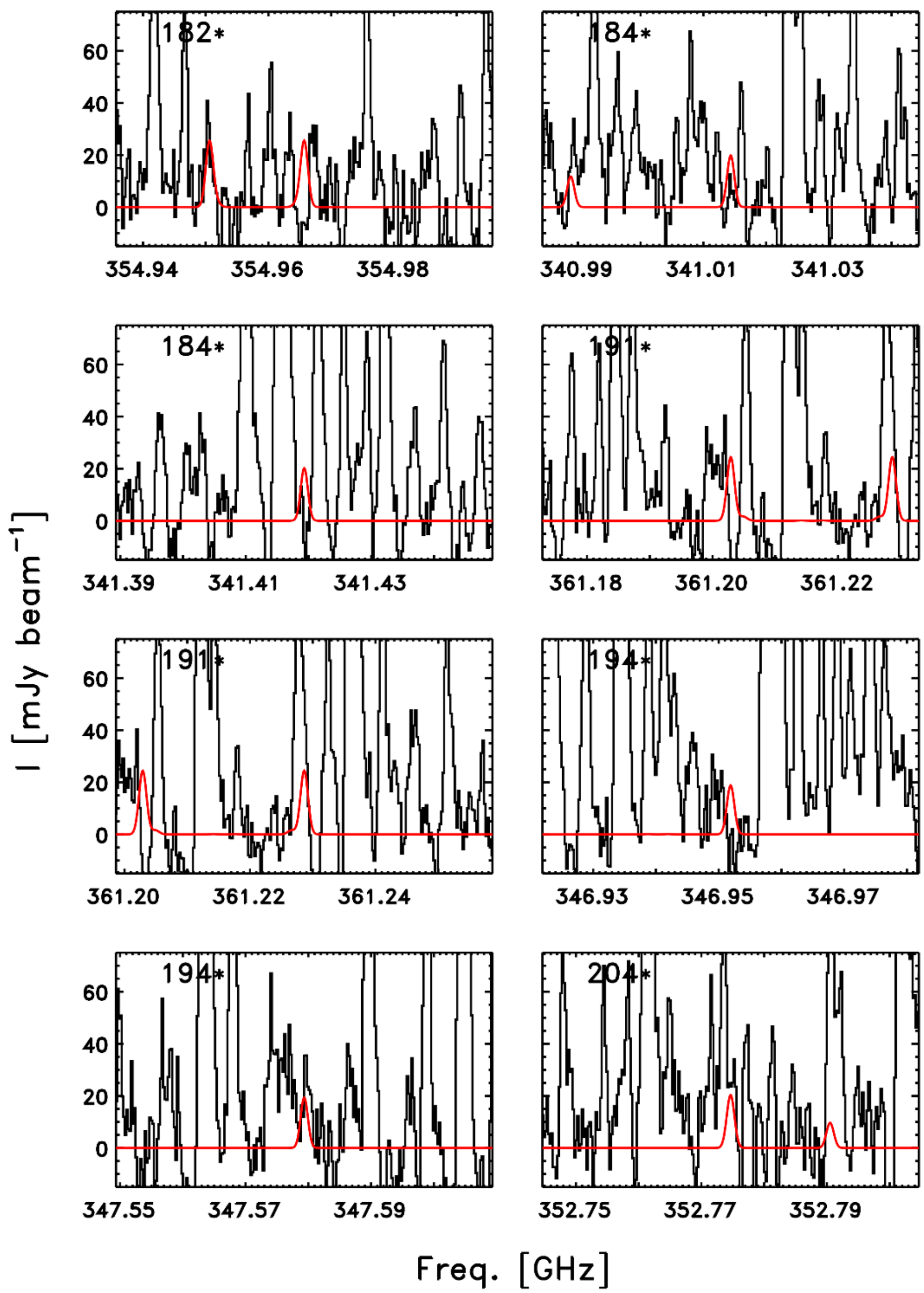

182*
184*
184*
191*
191*
194*
194*
204*
354.94
354.96
354.98
340.99
341.01
341.03
341.39
341.41
341.43
361.18
361.20
361.22
361.20
361.22
361.24
346.93
346.95
346.97
347.55
347.57
347.59
352.75
352.77
352.79
I [mJy beam^{-1}]
Freq. [GHz]

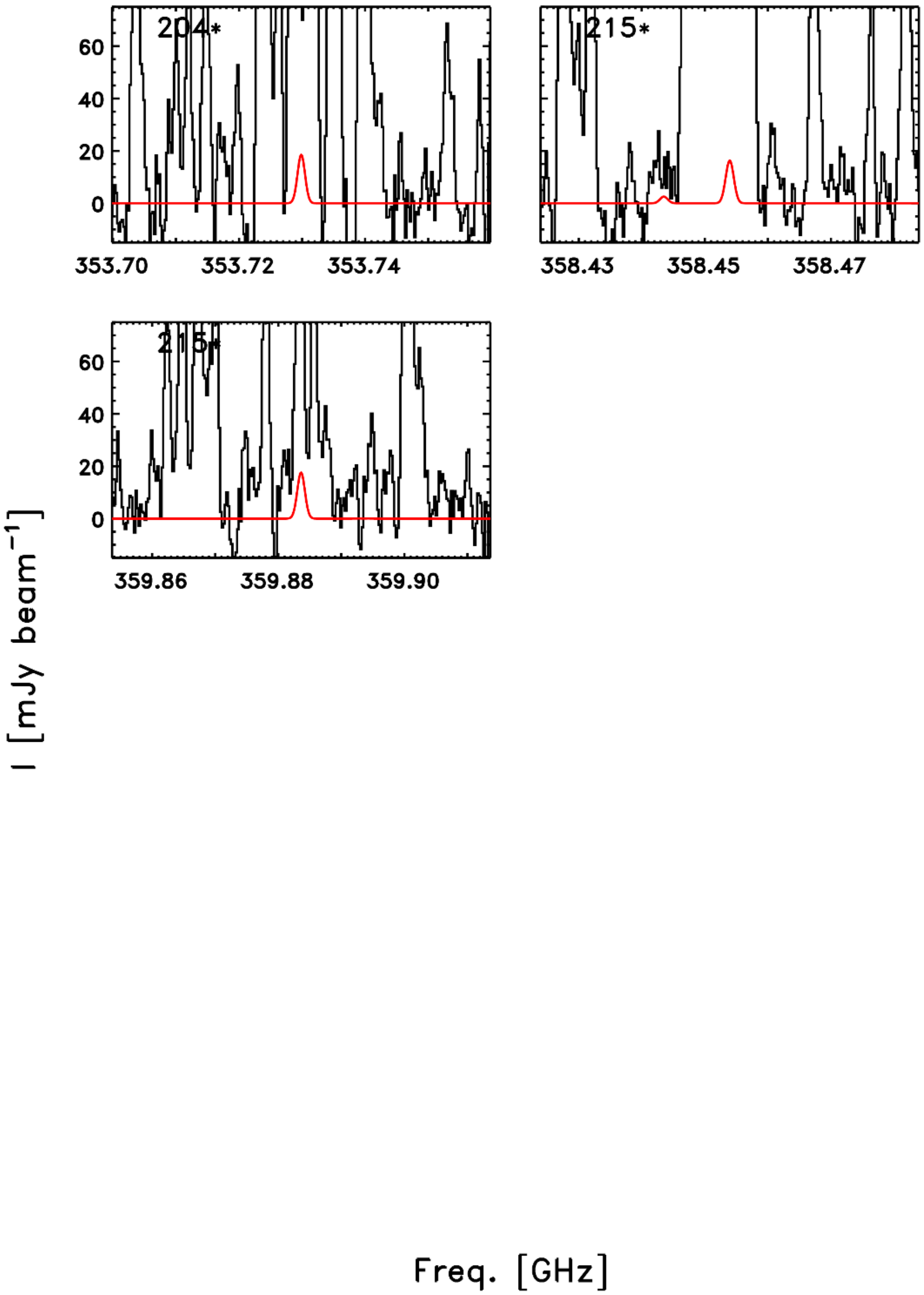

Figure S1: Search summary for strongly predicted 2-chloroethanol lines towards IRAS 16293.

Sources from Caltech Submillimeter Observatory (CSO)

In addition to the search towards the protostar IRAS 16293-2422B, the ^{35}Cl isotopologue of 2-chloroethanol was also sought in 29 different astronomical sources observed between September 2007 and June 2013 using the 10.4 m Leighton Telescope of the Caltech Submillimeter Observatory (CSO) with 230 GHz wideband receivers. Information on these sources and their classification can be found in the previously published description of this survey from Widicus Weaver *et al*.[1]

The 29 sources comprise a broad range of astrophysical environments relevant to interstellar chemistry, including high-mass hot cores, low-mass hot corinos, Class 0 protostars, protostellar outflows, HII regions, and the Galactic center shocked cloud G+0.693−0.027. The diversity of these sources provides a wide range of temperatures, densities, and evolutionary stages that may support the detection of 2-chloroethanol in the gas phase. Hot cores and hot corinos are particularly rich in complex organic molecules (COMs) due to thermal desorption of icy grain mantles, while Class 0 protostars and shocked regions provide insight into earlier stages of source evolution.

Despite this diversity, detections of chlorine-containing species in these sources remain limited. Hydrogen chloride (HCl) was first detected in the interstellar medium toward OMC-1 in Orion by Blake *et al*.[2] and was mapped across that region by Schilke *et al*.[3] HCl was also detected toward DR21(OH) in a comprehensive CSO survey of HCl[4] and toward L1157 B1 as part of the CHESS (Chemical Herschel Surveys of Star-forming regions) program using the Herschel Space Observatory.[5] In addition to HCl, chloronium (H_2Cl^+)[6] has been detected in absorption toward W51, however, the absorption arises primarily in foreground diffuse clouds unassociated with the hot core itself. The remaining 25 sources, to the best of our knowledge, have no reported chlorine-bearing molecule detections.

An initial search for the strongest predicted lines in the 29 previously analyzed sources was unsuccessful, using a prediction at 10K. All 29 sources were manually examined for possible matches of 2-chloroethanol transitions and low signal-to-noise lines in the observational spectra. While most of the sources yielded no coincident lines with 2-chloroethanol, GAL 010.47+00.03 appeared to have potential line matches. All of the potential line matches are heavily blended with transitions from other molecules or correspond with extremely low signal-to-noise spectral features. Therefore, no detection can be claimed for any of these 29 sources. A search for hydrogen chloride was intended to probe the chlorine chemistry of these sources; however, no rotational HCl transitions lie within the observed frequency range, and future studies are merited. It is important to note that the non-detections can be interpreted in two ways. The non-detection could result from a low abundance (or non-presence) of 2-chloroethanol in that source, or from beam dilution if the spatial distribution of 2-chloroethanol is compact compared to the single-dish beam of the CSO. In this case, because of the low abundance of Cl in the interstellar medium, the lack of detection of 2-cholorethanol is most likely due to its low abundance or non-presence.

The upper limits of the 2-chloroethanol column density in each source were then estimated using Global Optimization and Broadband Analysis Software for Interstellar Chemistry (GOBASIC),[7, 8] the details of which are described by Rad *et al*.[9] GOBASIC conducts a broadband spectral analysis by comparing the observational spectrum and spectral line catalog information. The analysis assumes a local thermodynamic equilibrium (LTE) and Gaussian line shapes. For these analyses, the δv was fixed at 0, and both T_{rot} and FWHM were constrained to the weighted average of corresponding values for all previously detected molecules[1] in each source, with the weighting set as the reciprocal of their estimated uncertainty. The results of the upper limit analysis are presented in Table S1.

Table S1. Estimation of column density upper limits for [^{35}Cl]-2-chloroethanol in the previously observed astronomical sources

source	type	T_{rot} (K)^a	FWHM (km s^{-1})^a	column density (cm^{-2})
W3(H_2O)	Hot Core	48.9	6.6	≤2.236 × 10^{14}
L1448 MM-1	Class 0 + Outflow	17.7	1.3	≤1.680 × 10^{11}
NGC 1333 IRAS 2A	Hot Corino	22.4	3.4	≤1.673 × 10^{12}
NGC 1333 IRAS 4A	Hot Corino	18.9	9.6	≤1.852 × 10^{13}
NGC 1333 IRAS 4B	Hot Corino	20.0	5.5	≤5.232 × 10^{13}
B1-b	Class 0	9.9	1.2	≤1.420 × 10^{13}
Orion-KL	Hot Core	80.6	9.4	≤4.206 × 10^{15}
NGC 2264	Hot Core	20.4	4.5	≤4.054 × 10^{13}
NGC 6334-29	Class 0	49.6	5.4	≤1.407 × 10^{14}
NGC 6334-38	Class 0	26.4	3.5	≤5.863 × 10^{13}
NGC 6334-43	Class 0	16.0	3.1	≤4.135 × 10^{13}
NGC 6334-IN	Class 0	20.2	3.9	≤4.403 × 10^{13}
GCM+0.693-0.027	Shocked Region	10^b	14.1^c	≤3.207 × 10^{14}
GAL 010.47+00.03	Hot Core	62.0	10.8	≤5.206 × 10^{14}
GAL 12.21-0.10	H II Region	28.9	8.2	≤4.956 × 10^{13}
GAL 012.91-00.26	Hot Core	29.4	4.9	≤3.206 × 10^{13}
HH 80/81	Outflow	19.0	2.4	≤1.521 × 10^{13}
GAL 19.61-0.23	Hot Core	53.7	9.0	≤8.712 × 10^{13}
GAL 24.33+0.11	Hot Core	18.2	4.7	≤1.803 × 10^{13}
GAL 24.78+0.08	Hot Core	38.0	7.2	≤6.353 × 10^{13}
GAL 31.41+0.31	Hot Core	79.1	7.4	≤1.287 × 10^{14}
GAL 34.3+00.2	Hot Core	45.8	6.8	≤2.112 × 10^{14}
GAL 45.47+0.05	Hot Core	15.1	4.6	≤1.660 × 10^{13}
W51	Hot Core	49.0	9.2	≤1.065 × 10^{14}
GAL 75.78+0.34	H II Region	28.0	4.2	≤1.537 × 10^{13}
W75N	Hot Core	42.7	5.6	≤2.053 × 10^{14}
DR21(OH)	Hot Core	39.0	6.0	≤1.516 × 10^{14}
L1157 MM	Class 0 + Outflow	14.3	5.8	≤1.130 × 10^{13}
NGC 7538	Hot Core	19.0	3.7	≤2.447 × 10^{13}

[a] The listed temperature and FWHM are the weighted average values from all other molecules fit in each source and were fixed during analysis.
[b] No temperature information is available. The temperature was assumed to be 10 K.
[c] FWHM of carbon sulfide

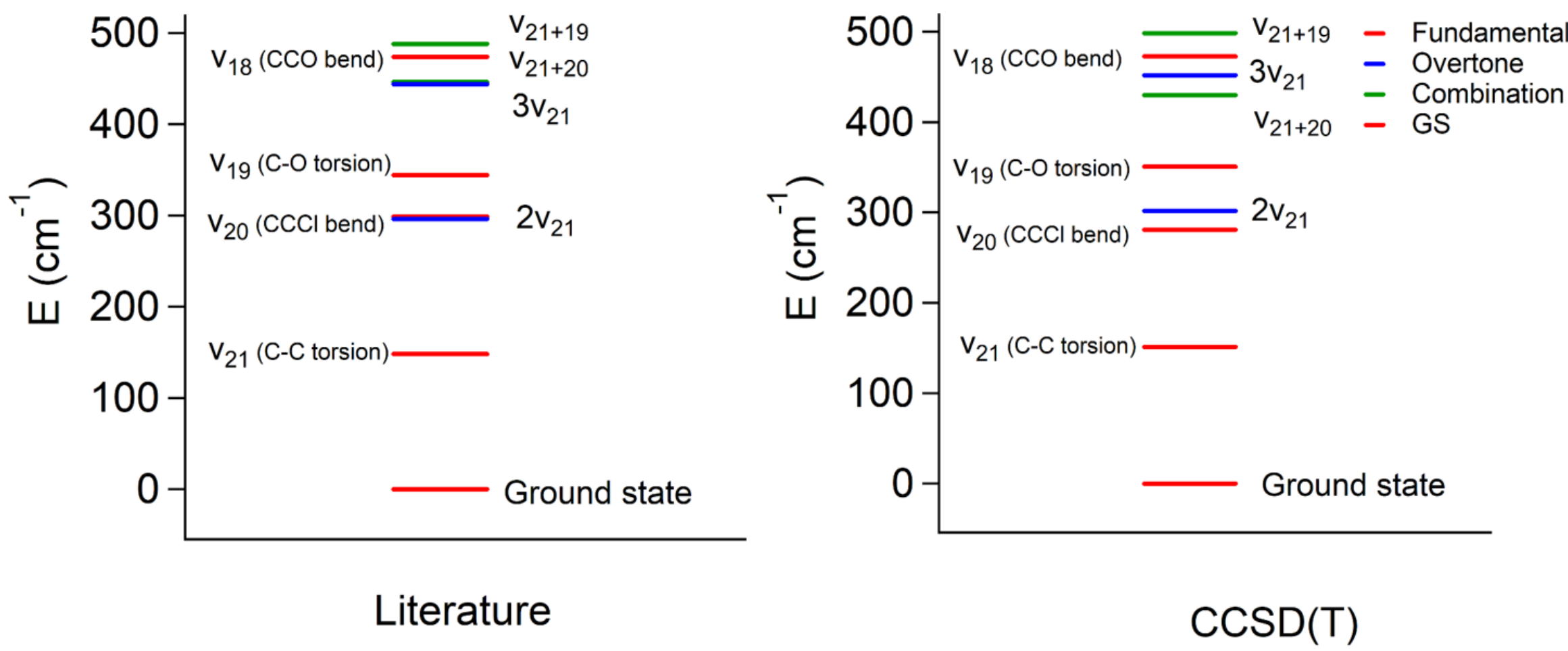

Figure S2: Energy levels of the vibrationally excited states of 2-chloroethanol reported in the literature (left) and CCSD(T) determined values (right). Note that overtones were not observed in the literature[10, 11] and so were extrapolated from the observed fundamental.

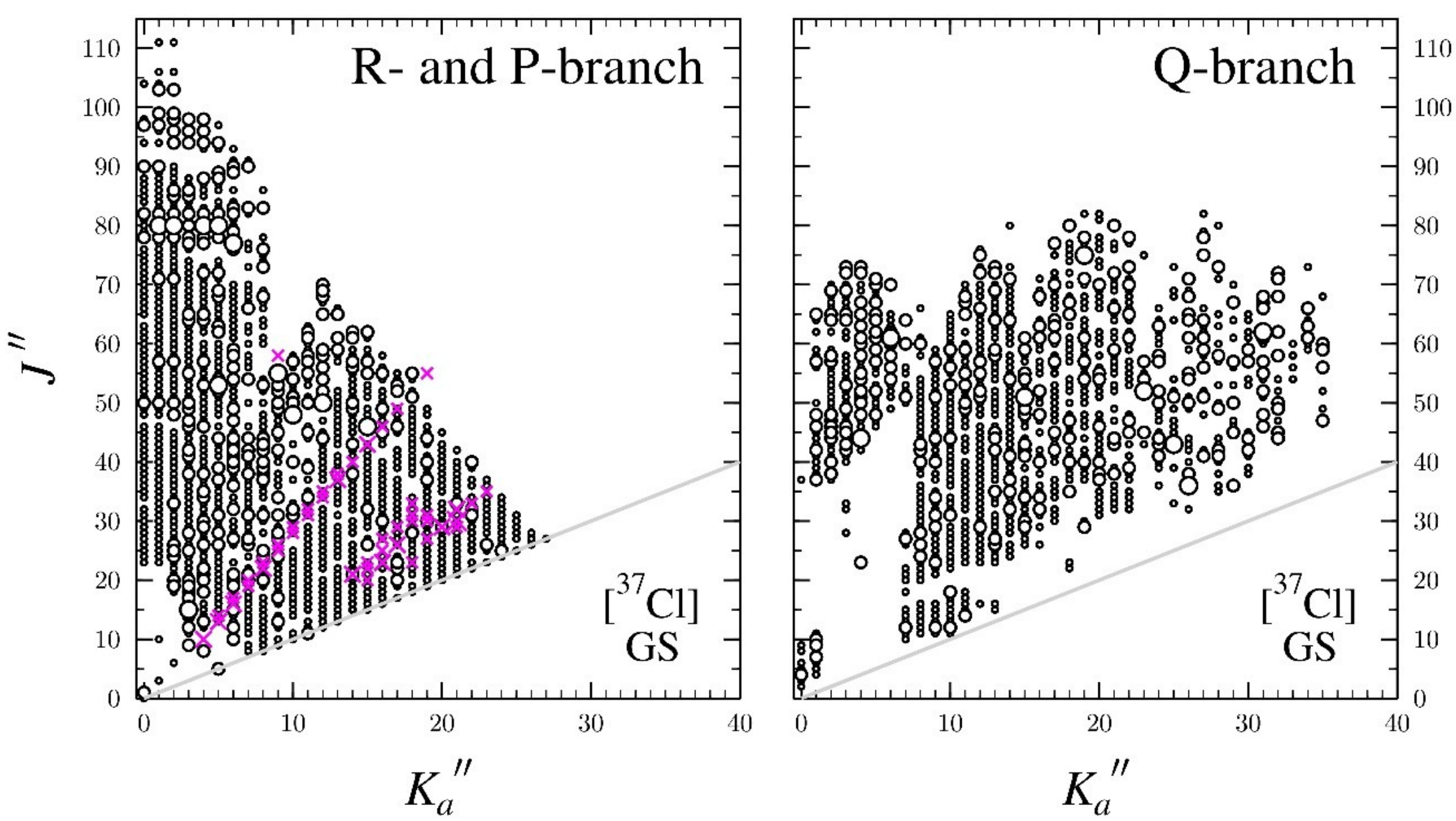

Figure S3. Data distribution plot for the least-squares fit of millimeter-wave spectroscopic data for the [^{37}Cl]-isotopologue of 2-chloroethanol, ground vibrational state, excluding hyperfine resolved transitions. The size of the outlined circle is proportional to the value of $|(f_{obs.} - f_{calc.})/\delta f|$, where δf is the frequency measurement uncertainty (50 kHz), and no quotient values are larger than three.

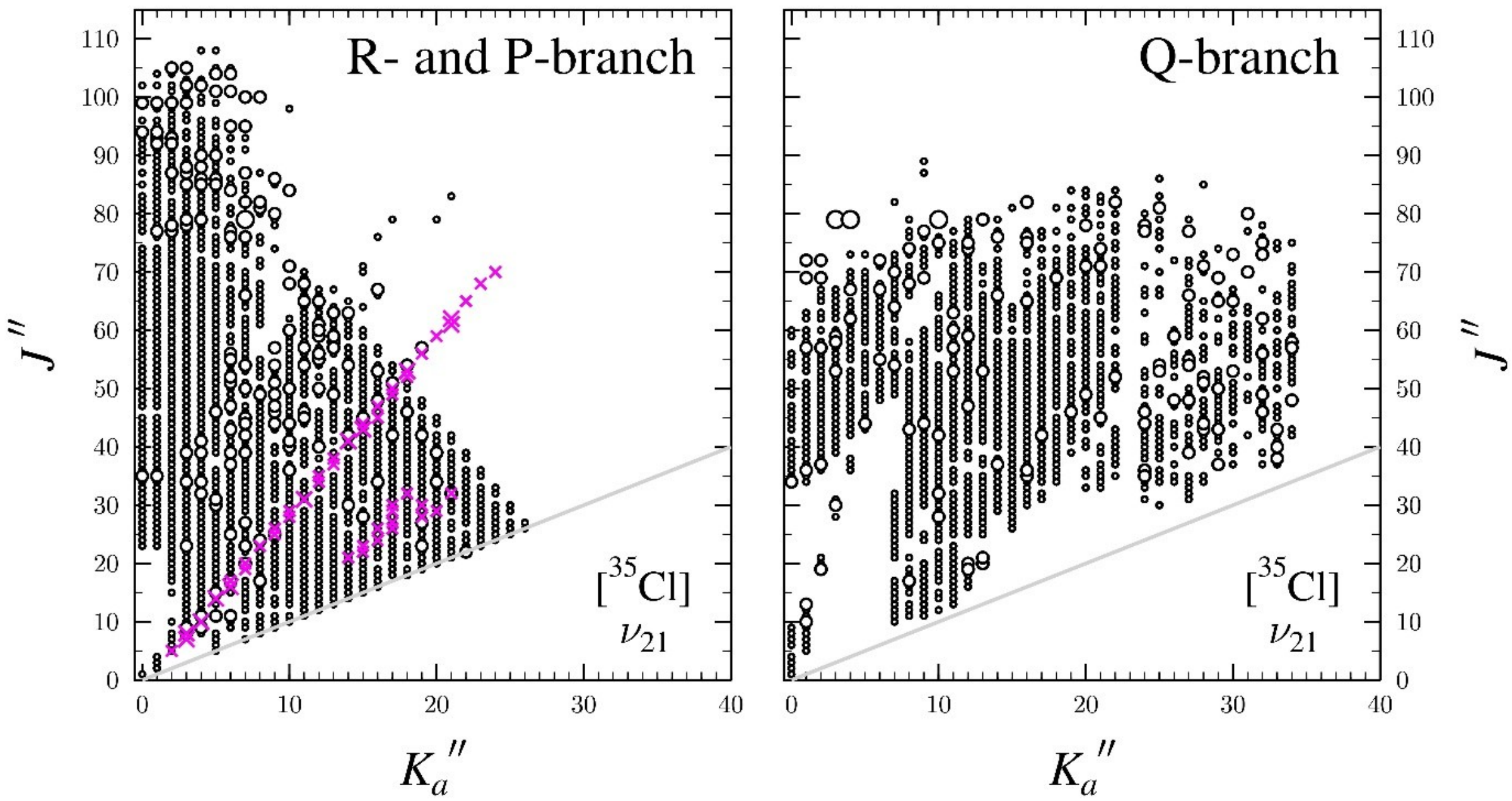

Figure S4. Data distribution plot for the least-squares fit of millimeter-wave spectroscopic data for the [^{35}Cl]-isotopologue of 2-chloroethanol, ν_{21}, excluding hyperfine resolved transitions. The size of the outlined circle is proportional to the value of $|(f_{obs.} - f_{calc.})/\delta f|$, where δf is the frequency measurement uncertainty (50 kHz), and no quotient values are larger than three.

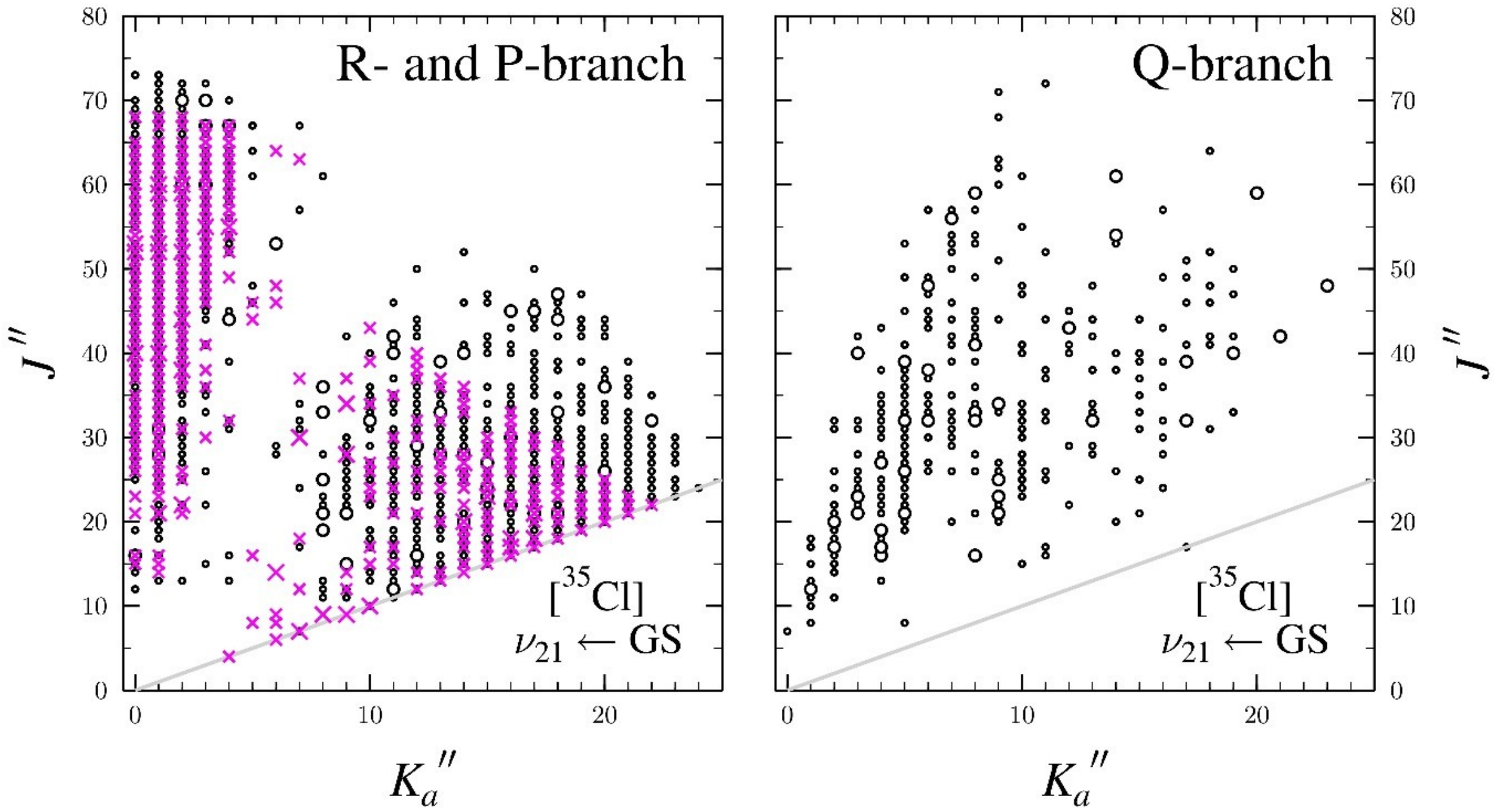

Figure S5. Data distribution plot for the least-squares fit of high-resolution infrared spectroscopic data for the [^{35}Cl]-isotopologue of 2-chloroethanol, ν_{21}, excluding hyperfine resolved transitions. The size of the outlined circle is proportional to the value of $|(f_{obs.} - f_{calc.})/\delta f|$, where δf is the frequency measurement uncertainty (50 kHz), and no quotient values are larger than three.

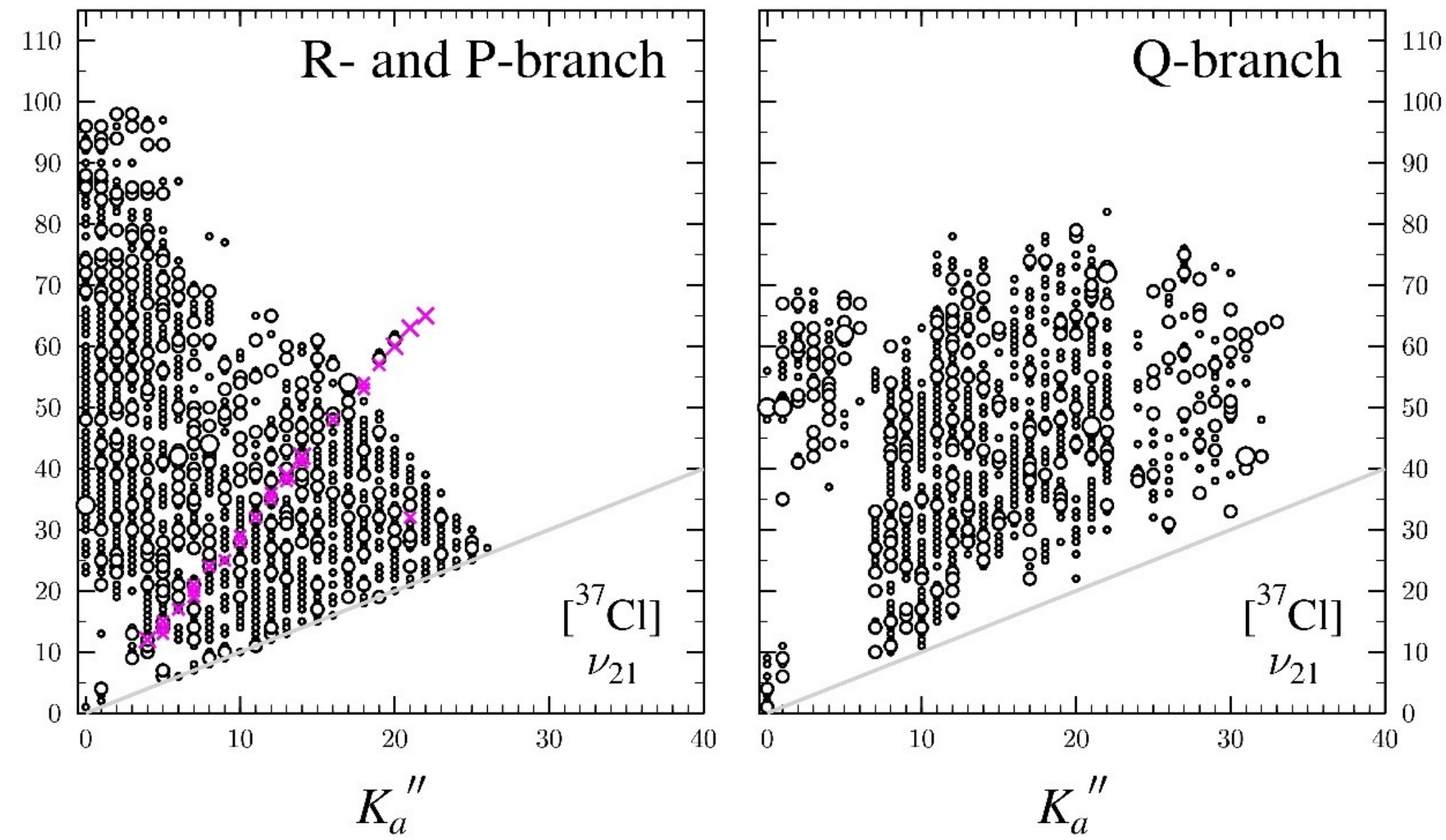

Figure S6. Data distribution plot for the least-squares fit of millimeter-wave spectroscopic data for the [^{37}Cl]-isotopologue of 2-chloroethanol, ν_{21}, excluding hyperfine resolved transitions. The size of the outlined circle is proportional to the value of $|(f_{obs.} - f_{calc.})/\delta f|$, where δf is the frequency measurement uncertainty (50 kHz), and no quotient values are larger than three.

References

(1) Widicus Weaver, S. L.; Laas, J. C.; Zou, L.; Kroll, J. A.; Rad, M. L.; Hays, B. M.; Sanders, J. L.; Lis, D. C.; Cross, T. N.; Wehres, N.; et al. Deep, Broadband Spectral Line Surveys of Molecule-rich Interstellar Clouds. *Astrophys. J., Suppl. Ser.* **2017**, *232*, 3. DOI: 10.3847/1538-4365/aa8098.

(2) Blake, G. A.; Keene, J.; Phillips, T. Chlorine in dense interstellar clouds-The abundance of HCl in OMC-1. *Astrophys. J.* **1985**, *295*, 501-506.

(3) Schilke, P.; Phillips, T.; Wang, N. Hydrogen chloride in OMC-1. *Astrophys. J.* **1995**, *441*, 334-342.

(4) Peng, R.; Yoshida, H.; Chamberlin, R. A.; Phillips, T. G.; Lis, D. C.; Gerin, M. A COMPREHENSIVE SURVEY OF HYDROGEN CHLORIDE IN THE GALAXY. *Astrophys. J.* **2010**, *723*, 218. DOI: 10.1088/0004-637X/723/1/218.

(5) Neufeld, D. A.; Black, J. H.; Gerin, M.; Goicoechea, J. R.; Goldsmith, P. F.; Gry, C.; Gupta, H.; Herbst, E.; Indriolo, N.; Lis, D.; et al. HERSCHEL OBSERVATIONS OF INTERSTELLAR CHLORONIUM. II. DETECTIONS TOWARD G29.96-0.02, W49N, W51, AND W3(OH), AND DETERMINATIONS OF THE ORTHO-TO-PARA AND 35Cl/37Cl ISOTOPIC RATIOS*. *Astrophys. J.* **2015**, *807*, 54. DOI: 10.1088/0004-637X/807/1/54.

(6) Lis, D. C. Herschel/HIFI line surveys: Discovery of interstellar chloronium (H_2Cl^+). In *AIP Conference Proceedings*, 2015; American Institute of Physics: Vol. 1642, pp 346-349.

(7) Wright, C. J.; Smith, R. N.; Kroll, J. A.; Shipman, S. T.; Widicus Weaver, S. L. Extending the Millimeter/Submillimeter Wave Spectrum of Ground State Pyruvic Acid for Comparison to Astronomical Data. *ACS Earth Space Chem.* **2022**, *6*, 482-495. DOI: 10.1021/acsearthspacechem.1c00353.

(8) Zou, L.; Widicus Weaver, S. L. Observation and Analysis of Interstellar Acetone in GAL 31.41+0.31, GAL 034.3+00.2, and GAL 10.47+00.03. *Astrophys. J.* **2017**, *849*, 139. DOI: 10.3847/1538-4357/aa8eea.

(9) Rad, M. L.; Zou, L.; Sanders, J. L.; Widicus Weaver, S. L. Global Optimization and Broadband Analysis Software for Interstellar Chemistry (GOBASIC). *Astron. Astrophys.* **2016**, *585*, A23.

(10) Durig, J.; Zhou, L.; Gounev, T.; Klaeboe, P.; Guirgis, G. A.; Wang, L.-F. Far infrared spectra, conformational equilibria, vibrational assignments and ab initio calculations of 2-chloroethanol. *J. Mol. Struct.* **1996**, *385*, 7-21.

(11) Soliday, R. M.; Bunn, H.; Sumner, I.; Raston, P. L. Far-Infrared Synchrotron Spectroscopy and Quantum Chemical Calculations of the Potentially Important Interstellar Molecule, 2-Chloroethanol. *J. Phys. Chem. A* **2019**, *123*, 1208-1216. DOI: 10.1021/acs.jpca.8b11333.
